# Au_42_(PET)_32_ Nanocluster Sensitizer Unlocks the Annihilator Potential of Rubrene, Enabling High‐Performance NIR‐to‐Visible Photon Upconversion

**DOI:** 10.1002/anie.202523868

**Published:** 2026-02-12

**Authors:** Masaaki Mitsui, Shinjiro Takano, Tatsuya Tsukuda

**Affiliations:** ^1^ Department of Chemistry College of Science Rikkyo University Tokyo Japan; ^2^ Department of Chemistry Graduate School of Science The University of Tokyo Tokyo Japan

**Keywords:** gold nanoclusters, near‐infrared, photon upconversion, triplet–triplet annihilation

## Abstract

Photon upconversion (UC) via triplet–triplet annihilation enables the conversion of near‐infrared (NIR) photons into visible light, offering opportunities for solar energy harvesting, photocatalysis, and biophotonics. However, progress has been limited by the lack of triplet sensitizers capable of fully exploiting rubrene, the representative annihilator/emitter for NIR‐to‐visible UC. Here, we report Au_42_(PET)_32_ (**Au_42_**; PET = 2‐phenylethanethiolate), a highly anisotropic, needle‐shaped gold nanocluster that unlocks the annihilator potential of rubrene, enabling high‐performance NIR‐to‐visible UC. The **Au_42_**/rubrene pair achieves record‐setting UC quantum yields (*Φ*_UC_, 50% maximum) of 16.5% (reabsorption‐corrected quantum yield *Φ*_UCg_ of 21.4%) with a low threshold intensity (*I*_th_) of 0.14 W cm^−2^ under 808 nm excitation and 12.3% (*Φ*_UCg_ = 15.0%) under 936 nm excitation—over two orders of magnitude higher than previously reported values above 850 nm. Quantitative analysis revealed a high spin‐statistical factor (*f* = 0.58) for rubrene, suggesting an attainable *Φ*_UC_ maximum of ∼30% in rubrene‐based systems. The remarkable performances arise from the unique electronic structure of **Au_42_**, which combines strong NIR absorption with high visible transparency, minimizing losses of the *S*_1_ annihilator and the UC photons. These findings establish **Au_42_** as a benchmark sensitizer and exemplify a design principle for realizing highly efficient, low‐threshold NIR‐to‐visible UC.

## Graphical abstract

A needle‐shaped gold nanocluster, Au_42_(PET)_32_, serves as a powerful triplet sensitizer, enabling rubrene to fully express its intrinsic annihilator capability (spin statistical factor *f* = 0.58). This sensitizer–annihilator pair achieves record‐high upconversion internal quantum yields of 21.4% (808 nm excitation) and 15.0% (936 nm excitation), showcasing the emerging role of gold nanoclusters in triplet–triplet annihilation photon upconversion.

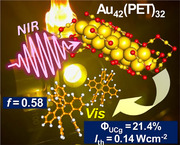

## 1 Introduction

Near‐infrared (NIR) light constitutes a significant fraction of the solar spectrum; however, most next‐generation photoactive systems—including perovskite and organic thin‐film solar cells as well as water‐splitting photocatalysts—are still unable to effectively utilize NIR photons owing to their limited or negligible light‐harvesting capabilities in this spectral region [1, 2, 3]. This fundamental constraint has stimulated growing interest in photon upconversion (UC) strategies, particularly triplet–triplet annihilation upconversion (TTA‐UC), which enables the conversion of low‐energy photons into higher‐energy emission even under low‐intensity, non‐coherent light conditions [4]. In TTA‐UC systems, rational pairing of triplet sensitizers with annihilators (emitters) is essential [5]. Owing to their versatility in combination, TTA‐UC has proven to be a highly adaptable approach for light‐energy conversion, allowing UC across a wide spectral range from the ultraviolet to the NIR depending on the chosen sensitizer–annihilator pair [5, 6, 7, 8, 9, 10, 11, 12, 13, 14, 15].

**SCHEME 1:**
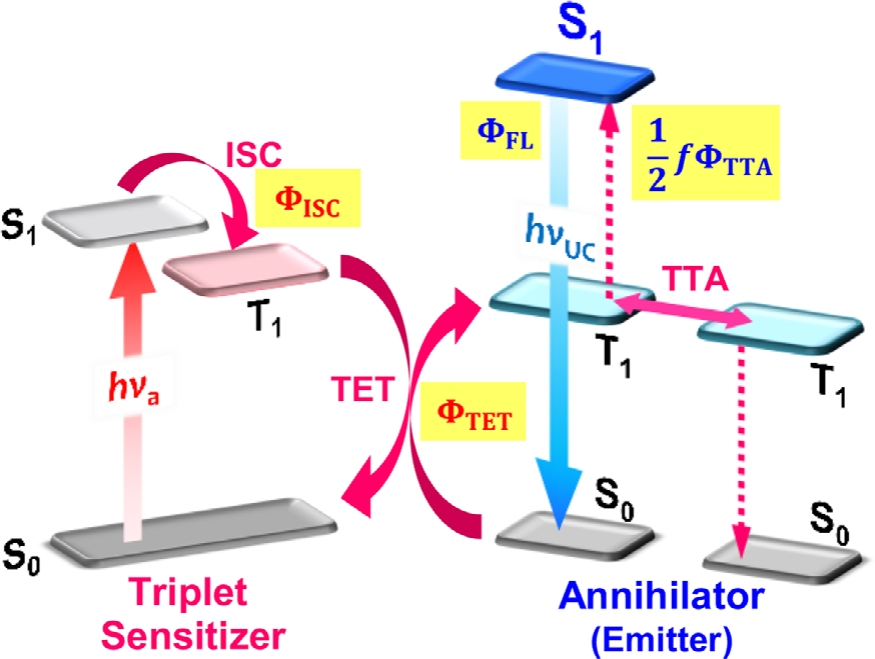
Mechanism of triplet–triplet annihilation photon upconversion (TTA‐UC), highlighting the quantum yields of the key photophysical processes.

As illustrated in Scheme 1, the TTA‐UC mechanism begins when a sensitizer absorbs a long‐wavelength photon (*hν*_a_) to populate an excited singlet state (*S*_1_), which undergoes intersystem crossing (ISC) to generate an excited triplet state (*T*_1_). This triplet energy is subsequently transferred to a ground‐state annihilator via diffusion‐mediated triplet energy transfer (TET). Finally, two triplet‐excited annihilators undergo TTA, generating an excited singlet annihilator that emits a higher‐energy photon (*hν*_UC_). Since TTA is a bimolecular (two‐photon) process, the theoretical maximum of TTA‐UC quantum yield (*Φ*_UC_) is 50%. It can be expressed as:

(1) $$\Phi_{\mathrm{UC}}=\frac{f}{2}\Phi_{\mathrm{ISC}}\Phi_{\mathrm{TET}}\Phi_{\mathrm{TTA}}\Phi_{\mathrm{FL}},$$

where *Φ*_ISC_, *Φ*_TET_, and *Φ*_TTA_ represent the quantum yields for ISC in the sensitizer, TET from the sensitizer to the annihilator, and TTA between annihilators, respectively. The *f* denotes the spin statistical factor, that is, the fraction of TTA events that generate an annihilator in the *S*_1_ state. *Φ*_FL_ is the fluorescence (FL) quantum yield of the annihilator in the UC sample solution. In addition to *Φ*_UC_, another key parameter defining TTA‐UC performance is the threshold excitation intensity (*I*_th_), which marks the transition of the excitation intensity dependence of UC emission from quadratic to pseudo‐linear behavior [16, 17]. From a practical standpoint, systems that exhibit both high *Φ*_UC_ and low *I*_th_ are highly desirable.

In visible‐to‐visible TTA‐UC, several sensitizer–annihilator pairs have achieved *Φ*_UC_ values exceeding 20% with *I*_th_ below 0.1 W cm^−2^ in the liquid phase [7, 18, 19, 20, 21, 22, 23]. Extending such performance to the NIR‐to‐visible regime, however, remains a significant challenge. Recently, *Φ*_UC_ values of 8%–10% have been reported in a few systems using semiconductor quantum dot (QD) sensitizers [24, 25]. However, most systems still exhibit significantly lower *Φ*_UC_ values in solution (Table S1) [19, 26, 27, 28, 29, 30, 31, 32, 33, 34, 35, 36, 37, 38, 39, 40, 41, 42, 43, 44, 45, 46, 47, 48, 49, 50]. The current benchmark InAs‐QD/rubrene system achieves *Φ*_UC_ = 10.6% and *I*_th_ = 20.2 W cm^−2^ under 808 nm excitation [25], which is still far from the best visible‐range performances. An effective NIR‐responsive sensitizer must combine strong NIR absorption, a high *Φ*_ISC_, and long triplet lifetimes, while minimizing loss pathways such as back TET from the annihilator, Förster resonance energy transfer (FRET) from the *S*_1_ annihilator, and reabsorption of upconverted photons. The creation of new sensitizers that meet these criteria would be a significant advancement in improving NIR‐to‐visible UC performance. On the annihilator side, high *f*, high *Φ*_FL_, and long triplet lifetimes are required. However, suitable annihilators for NIR‐to‐visible UC remain scarce, with rubrene being the most widely employed. Reported *f* values of rubrene vary considerably (0.155–0.60) [34, 35, 36, 50, 51, 52, 53], leaving the maximum achievable *Φ*_UC_ for rubrene‐based systems uncertain. To date, almost all rubrene‐based TTA‐UC systems have shown *Φ*_UC_ values below 8% (≤ *f*/2) [24, 25, 26, 27, 28, 29, 30, 31, 32, 33, 34, 35, 36, 37, 38], a limitation often attributed to the small *f* value (see Table S1) [34].

Recently, atomically precise metal nanoclusters (NCs) have emerged as promising triplet sensitizers, distinguished by efficient ISC (high *Φ*_ISC_) and microsecond‐scale triplet lifetimes [54, 55]. Their discrete energy levels and molecule‐like electronic structures, coupled with their tunable composition and surface ligation through core alloying or ligand exchange, enable precise control of both ISC and TET processes [54, 55, 56, 57, 58, 59, 60, 61, 62, 63, 64, 65, 66, 67, 68, 69, 70, 71, 72]. Nevertheless, the UC performance of NCs with spherical metal cores is inferior to that of visible‐to‐visible systems, as is the case with other sensitizers [22, 23, 57, 58, 59, 60, 61, 62, 63, 64, 65]. This shortfall stems from their limited NIR oscillator strengths and dominant absorption in the UV–visible region. Introducing anisotropic metal cores, in which the metallic framework extends along one dimension, offers an effective strategy to overcome this limitation. Recently, Jin and co‐workers developed a family of rod‐shaped gold NCs, Au_24+18_ *_n_* (PET)_20+12_ *_n_* (**Au_24+18_ *_n_***; PET = 2‐phenylethanethiolate; *n* = 1–5), whose cores consist of (4*n* + 2) layers of triangular Au_3_ units stacked in a hexagonal close‐packed arrangement [73, 74]. In these AuNCs, the highest occupied molecular orbital (HOMO) and lowest unoccupied molecular orbital (LUMO) are delocalized across the anisotropic metal core. This results in a pronounced reduction of the HOMO–LUMO gap and the emergence of a large transition dipole moment along the long axis of the core. More recently, they reported that the shortest member of this series, the Au_42_(PET)_32_ (**Au_42_**; *n* = 1) NC can serve as a triplet sensitizer for NIR‐to‐visible TTA‐UC when paired with the TES‐ADT (5,11‐bis(triethylsilylethynyl)‐anthradithiophene) annihilator [60]. However, the reported *Φ*_UC_ for this combination was modest, at 3.35% (out of a maximum of 50%), leaving significant room for improvement.

In this study, we elucidate the previously unexplored photophysical properties of the **Au_42_** sensitizer, including its *Φ*_ISC_, triplet energy (*E*_T_), and unique red‐edge excitation behavior, and demonstrate its full potential as a triplet sensitizer for NIR‐to‐visible UC. Combining **Au_42_** with rubrene affords *Φ*_UC_ values of 16.5% [reabsorption‐corrected value, that is, internal quantum yield *Φ*_UCg_, defined as the ratio of the number of UC photons generated to the number of photons absorbed by the sensitizer (#*hν*_UC_/#*hν*_a_), of 21.4%] under 808 nm excitation with a low *I*_th_ of 0.14 W cm^−2^, and 12.3% (*Φ*_UCg_ = 15.0%) under 936 nm excitation. These values were validated by accurately determining *Φ*_UC_ using the prompt FL of **Au_42_** as an internal reference. Furthermore, the results disclose a substantial spin statistical factor of rubrene (*f* = 0.58), establishing the **Au_42_**/rubrene pair as a new benchmark for NIR‐to‐visible UC systems.

## 2 Results and Discussion

### 2.1 Synthesis and Characterization

Details of the synthesis and characterization of **Au_42_** are provided in the Supporting Information. We adopted a recently developed controlled reduction method of Au(I)‐SR complexes to obtain highly anisotropic shapes [75, 76]. The method comprises two important points, including the use of sub‐stoichiometric amounts (4/5 eq.) of PET‐H ligand and reductant (1/6 eq.) relative to the Au(I) precursor and heat treatment of the crude product to obtain thermally stable products such as **Au_42_**. In the optimized condition, **Au_42_** can be synthesized in 12% yield based on Au after chromatographic separation. Its chemical identity and purity were examined by elemental analysis, mass spectrometry, and optical spectroscopy (Figure S1). These characterization results indicate that the obtained **Au_42_** is the same as that reported by Jin and co‐workers using a different synthesis method [74].

### 2.2 Photophysical Properties

**FIGURE 1:**
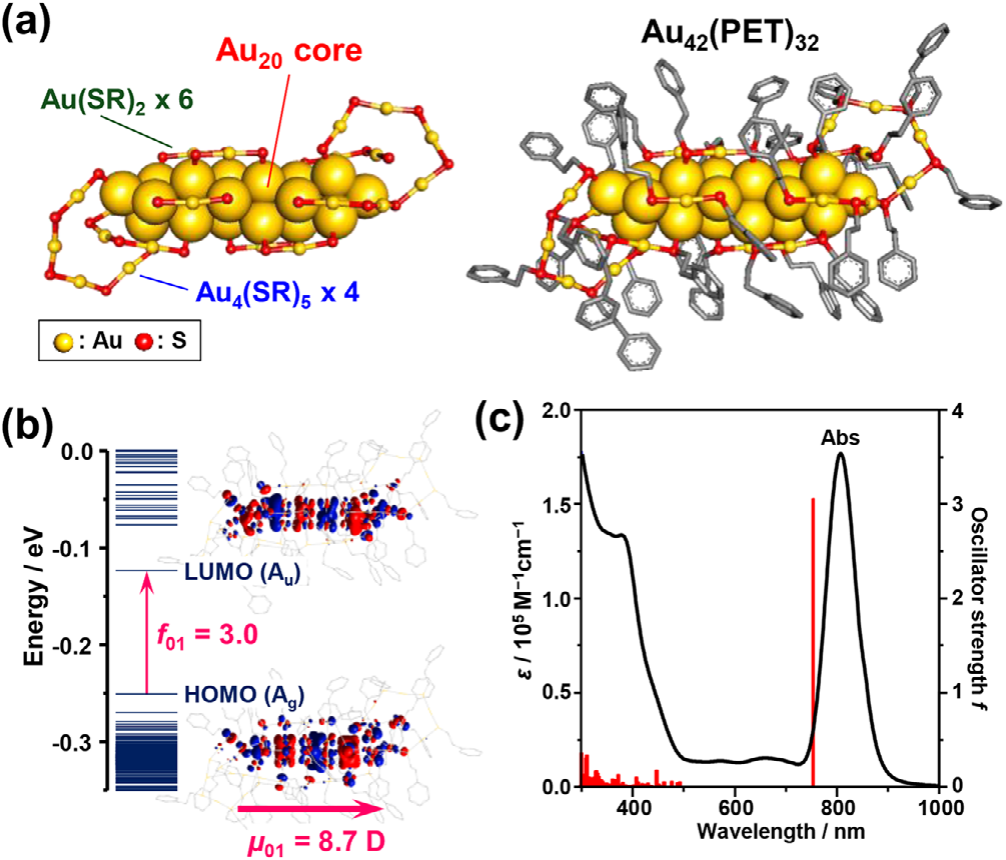
(a) Optimized structures of Au_42_(PET)_32_ (**Au_42_**; PET = 2‐phenylethanethiolate) obtained from DFT calculations. The right and left images show the structures with and without the Ph‐(CH_2_)_2_ moiety of PET, respectively (hydrogen atoms omitted). (c) The energy‐level diagram of the corresponding Kohn–Sham orbitals, including the oscillator strength (*f*_01_) and transition dipole moment (*μ*_01_) for the *S*_0_ → *S*_1_ (HOMO–LUMO) transition. (d) The experimental absorption spectrum of **Au_42_** in toluene compared with the TD‐DFT calculated stick spectrum.

Figure 1a shows the geometric structure of **Au_42_**. The **Au_42_** sensitizer features an Au_20_ core composed of six layers of triangular Au_3_ units stacked in a hexagonal close‐packed arrangement. This one‐dimensional Au_20_ core extension leads to pronounced delocalization of the HOMO (A_g_) and LUMO (A_u_) along the long axis, resulting in a significant reduction of the HOMO–LUMO gap (Figure 1b). Concomitantly, time‐dependent density functional theory (TD‐DFT) calculations for the optimized full structure of **Au_42_** (Figure S2) reveal an allowed *S*_0_(A_g_) → *S*_1_(A_u_) transition characterized by a pronounced transition dipole moment (*μ*_01_ = 8.7) and a large oscillator strength (*f*_01_ = 3.0), both predominantly oriented along the long axis of the Au_20_ core. These trends are consistent with the TD‐DFT results reported for Au_42_(SCH_3_)_32_, a simplified model of **Au_42_** [77, 78]. This anisotropic transition endows **Au_42_** with an intense, sharp S_0_–S_1_ absorption band centered near 800 nm, exhibiting a large molar extinction coefficient (*ε*_807_ = 1.75 × 10^5^ M^−1^ cm^−1^) in toluene (Figure 1c and Figure S3). In contrast, it retains relatively high optical transparency in the 500–750 nm visible region. The resulting UV–vis absorption spectrum (Figure 1c) is identical to that reported for structurally characterized Au_42_(S‐CH_2_Ph)_32_, thereby confirming the formation of the anisotropic Au_20_ core [73, 74]. Collectively, these optical properties establish **Au_42_** as a highly promising sensitizer for NIR‐to‐visible UC, as it enables efficient harvesting of approximately 800 nm photons while simultaneously suppressing reabsorption of UC emission and FRET from yellow/orange emitters such as rubrene and TES‐ADT (Figure S4).

**FIGURE 2:**
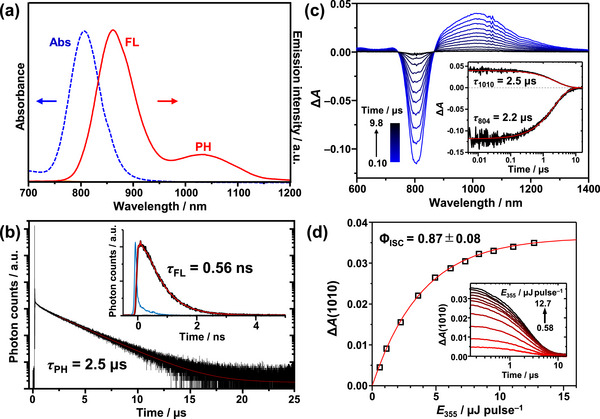
(a) Absorption (Abs) spectrum and emission spectrum comprising fluorescence (FL) and phosphorescence (PH) of **Au_42_** in deaerated toluene. (b) Time‐resolved FL and PH decay profiles of **Au_42_** (6µM). The fitting curve for the PH decay component is shown as a solid red line. Inset: FL decay curve (black) monitored at 880 ± 5 nm upon 805 nm excitation with the fitting curve (red) and the instrument response function (blue). (c) Transient absorption (TA) spectra of **Au_42_** (6 µM) in deaerated toluene recorded from 0.10 µs to 9.8 µs after 410 nm excitation. Inset: TA kinetic traces at 804 and 1010 nm, along with their corresponding fits. (d) Pump power dependence of the TA traces at 1010 nm (inset) for **Au_42_** (4 µM) in deaerated toluene, obtained 0.15 µs after the pump pulse. The solid line represents a least‐squares fit using Equation (S1).

As shown in Figure 2a, **Au_42_** in deaerated toluene displays dual emission: FL at 861 nm with *τ*_FL_ = 0.56 ns (*Φ*_FL_ = 5.0%) and phosphorescence (PH) at 1033 nm with *τ*_PH_ = 2.5 µs (*Φ*_PH_ = 2.3%) (Figure 2b and Table S2). The transient absorption (TA) spectra exhibit ground‐state bleaching (GSB) centered at 807 nm and broad excited‐state absorption (ESA) around 1010 nm (Figure 2c), both decaying with the PH lifetime, confirming their origin from the T_1_ state. To evaluate *Φ*_ISC_, the excitation‐intensity dependence of the ESA signal at 1010 nm was analyzed under 355 nm excitation using the partial saturation method (Figure 2d) [79], yielding *Φ*_ISC_ = 0.87 (±0.08). From *τ*_FL_ = 0.56 ns, the ISC rate constant (*k*_ISC_) was estimated to be 1.6 × 10^9^ s^−1^. As suggested by the theoretical analyses, this moderate rate is explained by the relatively small spin‐orbit coupling matrix elements (< 100 cm^−1^) associated with the direct *S*_1_ → *T*_1_ ISC and the absence of higher‐lying *T_n_* states [77, 78]. Nonetheless, the smaller FL radiative rate (*k*_r_^FL^ = *Φ*_FL_/*τ*_FL_ = 8.9 × 10^7^ s^−1^) and internal conversion rate (*k*_IC_ = [1–(*Φ*_FL_+*Φ*_ISC_)]/*τ*_FL_ = 1.4 × 10^8^ s^−1^) ensure efficient ISC, leading to the high *Φ*_ISC_ observed for **Au_42_**.

### 2.3 Triplet Sensitization

**FIGURE 3:**
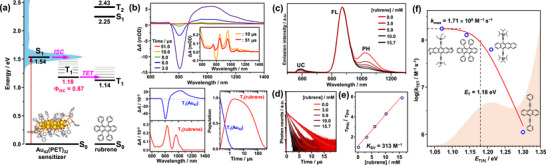
(a) Schematic energy‐level diagram illustrating the excited‐state relaxation pathways of **Au_42_**, including the TET process from **Au_42_** to rubrene. The numerical values indicate the energy levels of the excited states in eV relative to the ground state. (b) Time evolution of TA spectra of a deaerated toluene solution containing **Au_42_** (6 µM) and rubrene (15 mM), recorded at selected delay times (top). The inset shows TA spectra at 10 µs and 51 µs. The species‐associated spectra (SAS; bottom left) and corresponding time‐dependent concentration profiles (bottom right) of T_1_(Au_42_) and T_1_(rubrene) are also shown. (c) Emission spectra of **Au_42_** (6 µM) in toluene as a function of rubrene concentration. (d) Rubrene concentration dependence of the phosphorescence (PH) decay profiles of **Au_42_**, overlayed with the corresponding fitting curves. (e) Stern–Volmer plot constructed from the PH lifetimes in panel d, with a linear fit used to determine the Stern–Volmer constant (*K*_SV_) and the TET rate constant (*k*_TET_). (f) Logarithmic plot of *k*_TET_ for the **Au_42_** donor as a function of the *T*_1_ energy (*E*_T(A)_) of the aromatic acceptors. The solid line represents the fit based on the Sandros–Boltzmann equation.

In addition to a high ISC quantum yield (*Φ*_ISC_ = 0.87), achieving a *Φ*_TET_ close to unity is critically important for an effective triplet sensitizer (Figure 3a). To this end, we evaluated the ability of **Au_42_** to sensitize the triplet state of rubrene using TA spectroscopy. As shown in Figure 3b, the ESA decay of **Au_42_** is markedly accelerated upon the addition of 15 mM rubrene. Concomitantly, sharp ESA features corresponding to the vibronic progression of the rubrene *T*_1_ → *T*_2_ transition appear at 755, 846, and 960 nm (inset of Figure 3b); these features are entirely absent in the **Au_42_**‐only system. Although rubrene is known to exhibit strong triplet–triplet (*T*–*T*) absorption below 530 nm [53], measurements in this spectral region were precluded by the complete absorption of the probe light by the high concentration of ground‐state rubrene. Moreover, the weak *T*_1_ → *T*_2_ absorption of rubrene overlaps with the intense ESA of **Au_42_**, preventing direct observation of its rise dynamics. To overcome this limitation, we performed target analysis based on a kinetic model assuming TET from the *T*_1_ state of **Au_42_** to rubrene (see Supporting Information for details). This analysis yielded the species‐associated spectra (SAS; Figure 3b, bottom left) and the corresponding time‐dependent concentration profiles (bottom right) for the *T*_1_ states of **Au_42_** and rubrene. Both SAS faithfully reproduce the characteristic features of the experimental TA spectra, affording a TET rate constant of *k*_TET_ = 1.67 × 10^8^ M^−1^ s^−1^ and a rubrene *T*_1_ lifetime of 95 µs. These results unambiguously confirm the generation of rubrene triplets via sensitization by **Au_42_**.

As shown in the emission spectra (Figure 3c), progressive addition of rubrene selectively quenched the PH band and concomitantly reduced *τ*_PH_ (Figure 3d). The Stern–Volmer plot of *τ*_PH_ exhibited a linear dependence on rubrene concentration (Figure 3e), yielding a Stern–Volmer constant (*K*_SV_) of 313.2 (±10.7) M^−1^. The quenching rate constant (*k*_q_) was then calculated to be 1.25 × 10^8^ M^−1^ s^−1^ using the relation of *k*_q_ = *K*_SV_/*τ*_PH_. The *k*_q_ value thus estimated is in excellent agreement with the TA‐derived *k*_TET_ value of 1.67 × 10^8^ M^−1^ s^−1^. This strong consistency confirms that the PH quenching proceeds exclusively through TET, without involving any intermediate states, such as a charge‐transfer (CT) state. Therefore, the obtained *k*_q_ value (1.25 × 10^8^ M^−1^ s^−1^) corresponds to the intrinsic *k*_TET_. This conclusion is further supported by the large, positive Gibbs free energy changes (Δ*G*_CT_) calculated using the Rehm–Weller equation (Figure S5 and Table S3), which thermodynamically preclude any contribution from photoinduced CT processes.

It is noteworthy that the PH bands of metal NCs typically exhibit broad, featureless profiles lacking discernible vibronic structure. This makes it difficult to identify the exact position of the *T*_1_ → *S*_0_ origin transition. Consequently, accurate estimation of the *T*_1_ energy (*E*_T_) from PH spectra is challenging. To address this issue and further evaluate the triplet sensitization capability of **Au_42_**, Stern–Volmer analyses were performed for a series of acceptor molecules (Figure S6 and Table S4) [54]. Figure 3f plots log(*k*_TET_) against the *T*_1_ energy of each acceptor molecule, *E*_T(A)_. A pronounced increase in *k*_TET_ was observed with increasing driving force for TET, defined as Δ*E*_TET_ = *E*_T_—*E*_T(A)_. Once Δ*E*_TET_ exceeded zero, *k*_TET_ approached a nearly constant value, indicative of saturation behavior. This dependence confirms the involvement of the TET mechanism, which has been widely reported for both organic molecules [80] and metal NCs [23, 54, 62, 63], and can be quantitatively described by the Sandros–Boltzmann equation [80]:

(2) $$\log\left(k_{\mathrm{TET}}\right)=\log\left(\frac{k_{\max}}{1+\exp\left(-\frac{E_{T}-E_{T(A)}}{k_{B}T}\right)}\right)$$

where *k*_max_ represents the upper limit of *k*_TET_ for the investigated system, and *k*_B_ and *T* are the Boltzmann constant and absolute temperature, respectively. Fitting of the experimental data yielded an *E*_T_ value of 1.18 (±0.02) eV and a *k*_max_ of 1.71 × 10^8^ M^−1^ s^−1^ for **Au_42_**. The *T*_1_ energy derived in this manner coincided closely with the peak of the PH band (see Figure 3f). This agreement suggests that the maximum of the PH band corresponds closely to the *T*_1_ → *S*_0_ origin transition. This assignment is consistent with theoretical predictions that the structural rigidity of Au_42_(SCH_3_)_32_ leads to minimal geometric relaxation between these states [77, 78]. The broadening of the band around the 0–0 transition can be attributed to inhomogeneities associated with ligand conformations and the solvent environment, as well as strong electron–phonon coupling in **Au_42_** [81].

As shown in Figure 3a, the lowest triplet energy of **Au_42_** (1.18 eV) is slightly higher than that of rubrene (1.14 eV), providing an exothermic driving force of 0.04 eV for TET. This favorable alignment allows the **Au_42_**/rubrene pair to exhibit a *k*_TET_ value close to *k*_max_. However, the *k*_TET_ value (1.3 × 10^8^ M^−1^ s^−1^) is two orders of magnitude smaller than the estimated diffusion‐limited rate constant (*k*_d_) in toluene (*k*_d_ ∼1 × 10^10^ M^−1^ s^−1^) and falls within the typical range reported for metal NCs [54, 55]. This behavior can be explained by the localization of the hole and electron distributions of the *T*_1_ state primarily within the Au_20_ core, which is protected by the Au(I)‐PET staple motifs (Figure 1b). The Au(I)‐PET staples act as a steric barrier, hindering Dexter‐type TET. Metal NCs with similar core‐localized *T*_1_ states, such as [Au_25_(PPh_3_)_10_(PET)_5_Cl_2_]^2+^ [59], [Au_25–_ *_x_* Ag*_x_* (PPh_3_)_10_(PET)_5_Cl_2_]^2+^ [61], and [Au_25–_ *_x_* Cu*_x_* (PPh_3_)_10_(PET)_5_Cl_2_]^2+^ [59], exhibit comparable *k*_TET_ values (∼1 × 10^8^ M^−1^ s^−1^). Since *k*_TET_ for **Au_42_** with rubrene as the acceptor (*k*_TET_ = 1.3 × 10^8^ M^−1^ s^−1^) is moderate and the PH lifetime (2.5 µs) is not particularly long, it is essential to increase the acceptor concentration to achieve efficient TET. Increasing the rubrene concentration to ∼20 mM could raise the *Φ*_TET_ value to ∼0.9, enabling highly efficient triplet sensitization.

### 2.4 TTA‐UC Performance

**FIGURE 4:**
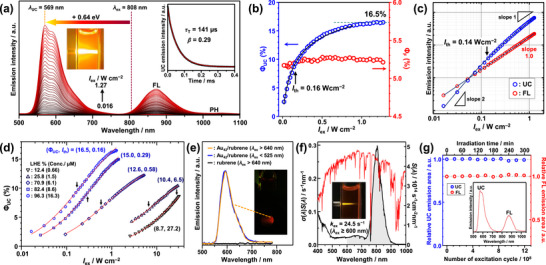
(a) Excitation‐intensity (*I*_ex_)‐dependent emission spectra of deaerated toluene containing **Au_42_** (16.3 µM) and rubrene (20 mM) under continuous‐wave 808 nm excitation. Inset: photograph under 808 nm laser irradiation without an optical filter. (b) UC quantum yield (*Φ*_UC_) and FL quantum yield (*Φ*_FL_), and (c) UC and FL intensities plotted as functions of *I*_ex_. (d) Dependence of *Φ*_UC_ and *I*_th_ on the concentration of **Au_42_** with 20 mM rubrene. Arrows indicate the *I*_th_ positions for each plot. (e) UC (orange) and prompt FL (blue) spectra of a deaerated toluene solution of **Au_42_**/rubrene under AM1.5G 1‐sun illumination, recorded using long‐pass (> 640 nm) and short‐pass (< 525 nm) filters, respectively. A rubrene‐only (20 mM) solution showed no emission upon > 640 nm excitation (black). Inset: photograph of the corresponding UC emission. Note that the FL spectra of rubrene differ markedly from that observed in a dilute solution due to strong reabsorption effects. (f) Spectrum of *σ*(*λ*)*S*(*λ*) and integrated values from 600 to 1000 nm, along with the global standard AM1.5G spectrum. Inset: photograph of the observed UC emission under irradiation with an 808 nm laser of *I*_ex_ = 1 mW cm^−2^. (g) Integrated UC and FL emission area as a function of excitation cycle number during continuous irradiation with an 808 nm laser (24 W cm^−2^) for a deaerated toluene solution containing **Au_42_** (16 µM) and rubrene (20 mM). The total irradiation time was 300 min. The vertical axes for UC and FL were offset to avoid overlap of data points. Inset: time‐dependent emission spectra recorded during continuous irradiation.

Figure 4a shows the emission spectra of a deaerated toluene solution containing **Au_42_** (16.3 µM) and rubrene (20 mM, nearly the saturation concentration in toluene [27]) under continuous‐wave (cw) 808 nm laser excitation. Dynamic light scattering measurements performed under these concentration conditions confirmed the absence of detectable aggregate formation (Figure S7). All UC measurements were conducted using an “edge‐excitation” geometry, in which the excitation beam was introduced through the cuvette edge to minimize reabsorption effects by the sensitizer and annihilator (Figure S8). Under these conditions, the UC emission from rubrene was observed at ∼570 nm, corresponding to an anti‐Stokes shift (Δ*E*_AS_) of 0.64 eV. The inset of Figure 4a displays the UC decay profile, which was fitted using Equation (S10) based on a kinetic model describing TTA between rubrene triplets. The extracted UC decay time of 141 µs and *β* parameter of 0.29 are consistent with the rubrene triplet lifetime (∼100 µs), as determined by TA measurements. The UC decay time is more than four orders of magnitude longer than the prompt FL lifetime of rubrene in toluene (14 ns) [82]. This fact provides definitive evidence that the observed UC emission originates from delayed FL via the TTA‐UC mechanism.

Figure 4b illustrates the excitation‐intensity (*I*_ex_) dependence of *Φ*_FL_ and *Φ*_UC_ of the **Au_42_**/rubrene system under 808 nm excitation. Notably, *Φ*_FL_ of **Au_42_** remained nearly constant across the entire intensity range, averaging 5.22% (±0.08) (Table S2). Furthermore, the FL lifetime of **Au_42_** (0.56 ns) remained unaffected by the addition of rubrene (Figure S9), confirming that its FL properties are retained in the UC environment. The invariance of the **Au_42_** FL properties validates the use of its FL band as an internal reference to accurately quantify the *Φ*_UC_. Such an internal reference allows for measurements under strictly identical conditions, including absorbance, solvent refractive index, excitation intensity, and optical geometry, and simplifies the evaluation to [83]:

(3) $$\Phi_{\mathrm{UC}}=\frac{S_{\mathrm{UC}}}{S_{\mathrm{FL}}}\Phi_{\mathrm{FL}}$$

where *S*_UC_ and *S*_FL_ represent the integrated UC and FL intensities, respectively (Figure S10). Notably, this evaluation method enabled highly reproducible acquisition of high‐quality UC data, as evidenced by the very small scatter of data points, as can be seen in Figure 4b–d. As shown in Figure 4b, the *I*_ex_‐dependence of *Φ*_UC_ exhibited a smooth increase followed by saturation, reaching 16.5%. This behavior was well reproduced using Equation (4), reported by Kamada and co‐workers [16]:

(4) $$\Phi_{\mathrm{UC}}=\left(1+\frac{1-\sqrt{1+4I_{\mathrm{ex}}/I_{\mathrm{th}}}}{2I_{\mathrm{ex}}/I_{\mathrm{th}}}\right)\Phi_{\mathrm{UC}}^{\infty }$$

Fitting yielded an *I*_th_ of 0.16 W cm^−2^. As shown in Figure 4c, the double‐logarithmic plot of FL intensity versus *I*_ex_ exhibited a linear dependence on *I*_ex_ with a slope of 1.0 across the entire measured range. In contrast, the UC intensity displayed the nonlinear behavior characteristic of TTA‐UC, with the slope changing from 2 to 1. This behavior was well reproduced by Equation (5) [16, 84]:

(5) $$I_{\mathrm{UC}}\propto K\left(1+\frac{1-\sqrt{1+4I_{\mathrm{ex}}/I_{\mathrm{th}}}}{2I_{\mathrm{ex}}/I_{\mathrm{th}}}\right)I_{\mathrm{ex}}$$

where *K* is an instrumental factor. Fitting with Equation (5) yielded an *I*_th_ of 0.14 W cm^−2^, which is essentially identical to the value (0.16 W cm^−2^) obtained from fitting Equation (4).

To optimize the UC performance of **Au_42_** in combination with rubrene, we investigated the dependence of *Φ*_UC_ and *I*_th_ on the light‐harvesting efficiency (LHE) of **Au_42_** at the photon detection position (Figure 4d and Figure S8). The LHE is herein defined as LHE = 1–10^–^ *^A^* ^(^ *^λ^* ^ex)/2^, where *A*(*λ*_ex_) denotes the absorbance of the sensitizer at the excitation wavelength (*λ*_ex_). As LHE increased, *Φ*_UC_ increased progressively while *I*_th_ decreased markedly, demonstrating clear enhancement of UC performance. At low LHE values, *I*_th_ was as high as 27.2 W cm^−2^ (LHE = 12.4%) and 6.5 W cm^−2^ (LHE = 25.8%). The limited output power of the excitation source prevented access to the saturation regime, resulting in low apparent *Φ*_UC_ values. However, with LHE exceeding 80%, the pronounced reduction in *I*_th_ enabled clear observation of *Φ*_UC_ saturation behavior (Figure 4b). The best performance, with an exceptionally high *Φ*_UC_ with a low *I*_th_, was obtained at the highest LHE of 96.3% (Figure 4a–g and Table 1).

**TABLE 1:** Photophysical parameters relevant to TTA‐UC for the Au_42_/rubrene pair in deaerated toluene.

Sensitizer	Annihilator	*λ*_ex_/nm	*Φ*_ISC_	*Φ*_TET_ ^a^	*f* ^b^	Δ*E*_AS_/eV	*Φ*_UC_	*Φ*_UCg_ (*Φ*_out_)^c^	*I*_th_/W cm^−2^ ^d^
**Au_42_**	Rubrene (20 mM)	808	0.87	0.86	0.58 ± 0.05	0.64	0.165	0.214 (0.775)	0.14 (0.16)
		936				0.86	0.123^e^	0.150 (0.820)	13.8 (13.3)
	TES‐ADT (10.1 mM)	808	0.87	0.81	0.35 ± 0.03^f^	0.59	0.044	0.092 (0.478)	1.17 (1.25)

^a^ Calculated using *K*_SV_[annihilator]/(1 + *K*_SV_[annihilator]).

^b^ Obtianed from the relationship described by Equation (1).

^c^ Internal quantum yield (50% maximum).

^d^ Values obtained from fitting with Equation (5); values in parentheses are those derived from Equation (4).

^e^ Not a saturated value.

^f^ *Φ*_FL_ = 0.74 taken from ref. [42] was used.

Due to these outstanding UC properties, distinct UC emission was clearly observed when the optimized **Au_42_**/rubrene solution was irradiated with a simulated AM1.5G 1‐sun spectrum (photograph in Figure 4e): the spectrum only included the spectral components above 640 nm, where rubrene exhibits negligible direct absorption. Under multicolor excitation conditions (*λ*_ex_ ≥ 600 nm), the excitation rate of the **Au_42_** sensitizer was calculated to be 24.5 s^−1^, corresponding to *I*_ex_ ≈ 10 mW cm^−2^ for monochromatic excitation at 808 nm. The photograph in Figure 4f shows that clear UC emission was also observed when the same solution was irradiated with 808 nm light at 1 mW cm^−2^. This further demonstrates the solar‐relevant operability of the **Au_42_**/rubrene system. In addition, the present system exhibits exceptional photostability (Figure 4g). Under continuous high‐intensity 808 nm irradiation at 24 W cm^−2^ (corresponding to an excitation rate of 6.7 × 10^4^ s^−1^), both UC and FL intensities retained more than 99% of their initial values after over 10^9^ excitation cycles per single NC (300 min of irradiation), and the absorbance of **Au_42_** at 808 nm remained unchanged before and after the measurement. Collectively, these results unequivocally demonstrate the high robustness of the **Au_42_**/rubrene pair under demanding excitation conditions.

**FIGURE 5:**
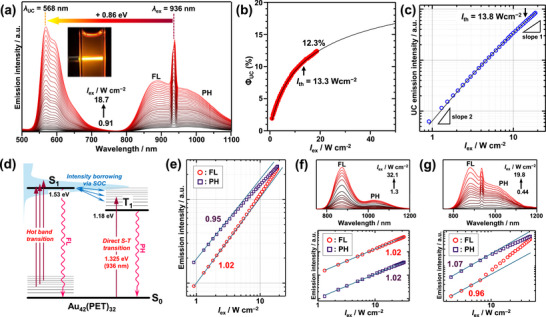
(a) Dependence of the emission spectra of deaerated toluene solutions containing **Au_42_** (16.3 µM) and rubrene (20 mM) on excitation intensity (*I*_ex_) under continuous‐wave 936 nm excitation. Inset: photograph of the same solution under 936 nm laser irradiation without an optical filter. (b) UC quantum yield (*Φ*_UC_) and (c) UC emission intensity plotted as functions of *I*_ex_. (d) A schematic energy‐level diagram illustrating the *S*_0_ → *S*_1_ hot‐band transition and the *S*_0_ → *T*_1_ transition in **Au_42_**. (e) *I*_ex_‐dependence of the fluorescence (FL) and phosphorescence (PH) intensities of **Au_42_** in the same mixed solution. (f, g) *I*_ex_‐dependent FL and PH spectra and intensities for a deaerated toluene solution of **Au_42_** (15.4 µM) alone under (f) 808 nm and (g) 936 nm excitation.

Figure 5a shows the *I*_ex_‐dependence of the emission spectra obtained under cw 936‐nm excitation for the same **Au_42_**/rubrene solution used in Figure 4a. At 936 nm, the LHE at the photon detection position was only 2.3% due to the small molar absorption coefficient (*ε*_936_ = 2,340 M^−1^ cm^−1^), significantly smaller than that at 808 nm (96.3%). Despite the low LHE, a *Φ*_UC_ of at least 12.3% was achieved (Figure 5b and Figure S11). Extrapolation of the fit using Equation (4) suggests that the saturated *Φ*_UC_ value would approach that obtained under 808 nm excitation (∼17%). Under this red‐edge excitation condition, the *I*_th_ value was 13–14 W cm^−2^ (Figure 5b,c), which is markedly higher than that for 808 nm excitation and reflects the significantly lower LHE at 936 nm. This trend is consistent with the relationship [16]:

(6) $$I_{\mathrm{th}}\propto \frac{1}{\mathrm{LHE}\cdot \Phi_{\mathrm{ISC}}\Phi_{\mathrm{TET}}k_{\mathrm{TTA}}\tau_{T(A)}^{2}}$$

where *k*_TTA_ is the bimolecular rate constant for the TTA process and *τ*_T(A)_ is the triplet lifetime of the annihilator.

Excitation at 936 nm (1.32 eV) corresponds to the *S*_1_–*T*_1_ energy gap region of **Au_42_**, where vibrationally excited *T*_1_ levels are directly accessible. As illustrated in Figure 5a, this excitation yields intriguing emission behaviors, including clear FL despite sub‐*S*_1_ excitation energy and a markedly intensified PH band. Figure 5d shows that the FL likely arises from hot‐band transitions from vibrationally excited *S*_0_ levels to *S*_1_, while the PH reflects direct *S*_0_ → *T*_1_ transitions. These direct *S*–*T* transitions are enabled by intensity borrowing from the strongly allowed *S*_0_ → *S*_1_ transition via SOC, which becomes particularly prominent near the *S*_1_ energy region [85]. Accordingly, the long‐wavelength tail of the first absorption band of **Au_42_** can be ascribed to triplet‐state contributions through strong *S*–*T* mixing. These transitions allow for the direct population of the T_1_ state, which is consistent with the distinct *I*_ex_‐dependencies of FL and PH under 808 nm and 936 nm excitation (Figure 5e–g). Under 808 nm excitation, both emissions increase linearly with *I*_ex_ (slope = 1.02), whereas 936 nm excitation yields superlinear FL and sublinear PH dependencies. This reciprocal behavior is more pronounced in the **Au_42_**‐only solution (Figure 5f,g), suggesting that TTA between two **Au_42_** triplets becomes active at *I*_ex_ above ∼10 W cm^−2^. This process can enhance the delayed FL contribution, as shown in Figure S12. These results demonstrate that **Au_42_** itself possesses an intrinsic capability for TTA‐UC, highlighting its unique photophysical properties. However, it should be emphasized that this self‐TTA pathway remains a minor process in the **Au_42_**/rubrene system. Given the extremely low concentration of **Au_42_** relative to rubrene (20 mM), together with the excellent agreement between the experimental data and Equations (4) and (5), which do not include loss terms (Figure 5b,c), we conclude that self‐TTA of **Au_42_** under 936 nm excitation does not significantly affect the overall TET and UC quantum yields.

**FIGURE 6:**
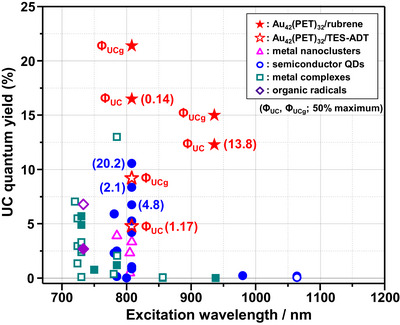
Comparison of UC quantum yields for **Au_42_** with those reported for various sensitizers used in NIR‐to‐visible UC systems, including metal nanoclusters, semiconductor QDs, metal complexes, and organic radicals under excitation wavelengths above 700 nm. Filled symbols represent data obtained with rubrene annihilators [24, 25, 26, 27, 28, 29, 30, 31, 32, 33, 34, 35, 36, 37, 38], whereas open symbols correspond to other annihilators [19, 39, 40, 41, 42, 43, 44, 45, 46, 47, 48, 49, 86]. The values in parentheses indicate the threshold intensities (in W cm^−2^). Detailed experimental conditions, including solvents and annihilator concentrations for each system, are summarized in Table S1.

Figure 6 compares the *Φ*_UC_ values of the **Au_42_**/rubrene system determined in this study with those of other NIR‐to‐visible UC systems reported previously. However, direct comparisons of the *Φ*_UC_ values across different systems reported in literature are not straightforward because reabsorption behavior in concentrated annihilator solutions depends sensitively on the measurement setup (Figure S8) [34]. To make a fair comparison with the related **Au_42_**/TES‐ADT system, we measured its UC performance using the optical setup and analysis procedures employed in this study. We determined the *Φ*_UC_ value to be 4.4% (Figure S13), which is higher than the value determined by Jin and co‐workers (3.4%) using a center‐excitation setup [60]. Under 808 nm excitation, the **Au_42_**/rubrene system achieved a *Φ*_UC_ of 16.5% with a remarkably low *I*_th_ of 0.14 W cm^−2^, far exceeding the previous benchmark of the InAs‐QD/rubrene pair (*Φ*_UC_ = 10.6% and *I*_th_ = 20.2 W cm^−2^) [25]. More strikingly, under 936 nm excitation, the **Au_42_**/rubrene pair demonstrated a transformative advance, delivering *Φ*_UC_ values of 12.3% even before reaching saturation. This performance dramatically surpasses those of all previously reported NIR‐to‐visible UC systems operating beyond 850 nm, where *Φ*_UC_ values have remained below 0.1%. To facilitate comparison of the *Φ*_UC_ values obtained with different setups, we estimated the reabsorption‐corrected *Φ*_UC_ values (*Φ*_UCg_) using the procedure described in the Supporting Information (Figure S10). The *Φ*_UCg_ values for the **Au_42_**/rubrene pair reached 21.4% and 15.0% under excitation at 808 and 936 nm, respectively.

### 2.5 Insight Into Spin Statistical Factor of Rubrene

The spin statistical factor (*f*) is an intrinsic property of the annihilator molecule that imposes a theoretical upper limit on *Φ*_UC_ when all other processes approach unity efficiency, expressed as *Φ*_UC_ ≤ *f*/2. Reported *f* values of rubrene vary considerably (0.155–0.60), leaving the maximum achievable *Φ*_UC_ for rubrene‐based systems uncertain [34, 35, 36, 50, 51, 52, 53]. Kazlauskas et al. estimated *f* = 0.155 from steady‐state measurements, suggesting that the generally low *Φ*_UC_ values in rubrene‐based systems stem from this small *f* value [34]. Conversely, Schmidt et al. reported *f* ≈ 0.6 based on pulsed‐laser measurements [50, 51], and Bossanyi et al. rationally explained this high value as arising from a reverse ISC channel activated by the favorable energy level alignment *S*_1_ (2.25 eV) < 2T_1_ (1.14 eV × 2) < T_2_ (2.43 eV) and the formation of weakly exchange‐coupled triplet pair states [53]. In this study, the *f* value of rubrene was experimentally determined using the reabsorption‐corrected UC quantum yield (*Φ*_UCg_) obtained by the internal reference method (Figure S10). To ensure reliability, each parameter in Equation (1) was independently and rigorously determined: (i) *Φ*_ISC_ = 0.87 (±0.08) was obtained for **Au_42_** using a partial saturation method based on the excitation‐intensity dependence of the ESA signal (Figure 2d); (ii) *Φ*_TET_ = 0.86 (±0.01) was calculated from the Stern‐Volmer constant under UC conditions (Figure 3e); (iii) *Φ*_TTA_ ∼1 was confirmed by the linear power dependence of UC emission at the measurement intensity, signifying the TTA‐saturation regime (Figure 4c). When *Φ*_UC_ in Equation (1) is replaced by *Φ*_UCg_, which corrects for inner‐filter effects, the *Φ*_FL_ can be taken as that of a highly dilute rubrene solution (0.98) [87] rather than the value in the UC solution. Using *Φ*_UCg_ = 0.214 ± 0.001 together with the determined parameters above, the spin statistical factor was calculated to be *f* = 0.58 ± 0.05. This value is in excellent agreement with that reported by Schmidt et al. and strongly suggests that rubrene annihilators are ideally capable of achieving *Φ*_UCg_ values approaching ∼30%. Accordingly, the realized *Φ*_UCg_ of 21.4% (corresponding to *Φ*_UC_ = 16.5%) represents a significant leap toward the theoretical efficiency limit of rubrene‐based TTA‐UC systems.

### 2.6 Origin of High‐Performance TTA‐UC

Understanding why the **Au_42_**/rubrene pair exhibits a markedly higher *Φ*_UC_ than systems employing metal complexes or semiconductor QD sensitizers with rubrene [24, 25, 26, 27, 28, 29, 30, 31, 32, 33, 34, 35, 36, 37, 38] provides profound insight into the factors that govern NIR‐to‐visible UC performance. A key experimental observation is that the *Φ*_FL_ of **Au_42_** remains constant under intense UC emission, which confirms negligible FRET from *S*_1_‐rubrene to **Au_42_**. This finding is supported by quantitative estimates: the calculated Förster radius (*R*_0_) for rubrene‐to‐**Au_42_** FRET is 4.7 nm (Figure S14a). Assuming a homogeneous distribution of the *S*_1_ annihilators generated via TTA and **Au_42_** (16 µM), the average nearest donor–acceptor distance (*R*_DA_) is estimated to be ∼22 nm, where the FRET efficiency is negligible (< 0.01%; Figure S14b). In contrast, semiconductor QDs such as PbS exhibit an extremely strong visible absorption coefficient (∼10^6^ M^−1^ cm^−1^) [88], which significantly overlaps with the FL spectrum of rubrene. Accordingly, the *R*_0_ values of QDs are anticipated to approach *R*_DA_, potentially giving rise to non‐negligible *S*_1_‐annihilator losses via FRET. These comparisons highlight the unique optical advantage of the **Au_42_**/rubrene system: strong NIR absorption combined with high visible transparency (Figure S4), which effectively suppresses FRET losses even under near‐unity LHE.

Another possible loss pathway is CT from *S*_1_‐rubrene to **Au_42_**. Thermodynamic analysis based on the redox potentials of **Au_42_** and rubrene (Table S3) yields Δ*G*_CT_ values of –0.18 eV for electron transfer and +0.29 eV for hole transfer, indicating that only the former is marginally favorable. However, at the low sensitizer concentration used (≤ 16 µM), even assuming diffusion‐limited kinetics (∼10^10^ M^−1^ s^−1^), the effective CT rate (∼10^5^ s^−1^) is several orders of magnitude smaller than the radiative decay rate of rubrene (∼10^8^ s^−1^). Consistently, *Φ*_UC_ increases with **Au_42_** concentration (Figure 4d), confirming that CT does not play a significant role in UC emission quenching.

Equally importantly, the high visible transparency of **Au_42_** strongly suppresses reabsorption of UC photons caused by the inner‐filter effect, allowing rubrene FL in the 550–600 nm region to be transmitted without attenuation (Figure S4). This unique optical window enables near‐unity NIR LHE while minimizing visible UC photon losses. In contrast, other NIR‐absorbing AuNCs, such as [Au_25_(PET)_18_]^−^ and [Au_25_(PPh_3_)_10_(PET)_5_Cl_2_]^2+^ (Au_25_‐rod), exhibit nearly zero transmittance (<10^−6^) in the visible region at comparable NIR LHE (Figure S4b), making efficient UC incompatible with high LHE. Therefore, the simultaneous realization of high *Φ*_UC_ and low threshold intensity (*I*_th_) in the **Au_42_**/rubrene system originates from the combination of nearly complete NIR absorption and exceptional visible transparency.

Equation (6) shows that *I*_th_ is inversely proportional to the square of the annihilator triplet lifetime (*τ*_T(A)_). This indicates that long‐lived annihilator triplets are necessary to achieve a low‐threshold for TTA‐UC. For example, 9,10‐diphenylanthracene (DPA), a benchmark visible‐range annihilator with long triplet lifetimes (several ms), achieves an ultralow *I*_th_ value (∼1 mW cm^−2^) [7, 23, 89]. However, annihilators for NIR‐to‐visible UC, such as rubrene and TES‐ADT, possess much shorter triplet lifetimes (∼0.1 ms; Figure 4a and Figure S13a). These lifetimes are over an order of magnitude shorter than that of DPA, due to rapid nonradiative T_1_ → S_0_ decay governed by the energy‐gap law. Recognizing this intrinsic limitation, this study pursued reducing *I*_th_ by maximizing the sensitizer's LHE instead. Unlike conventional sensitizers, which suffer from back TET or FRET losses at high concentrations, **Au_42_** enables efficient upconverted photon generation without such parasitic pathways. Consequently, as shown in Figure 4d, increasing the **Au_42_** concentration to achieve near‐unity LHE simultaneously realized high *Φ*_UCg_ (21.4%) and low *I*_th_ (0.14 W cm^−2^). However, this intensity threshold is still approximately two orders of magnitude higher than that of high‐performance visible‐to‐visible UC systems (∼1 mW cm^−2^). Closing this gap will require continued efforts in molecular design to further extend the triplet lifetimes of annihilators for NIR‐to‐visible UC.

## 3 Conclusion

In conclusion, this study establishes the **Au_42_**/rubrene system as a new benchmark for NIR‐to‐visible TTA‐UC. Leveraging the unique photophysical attributes of the structurally anisotropic **Au_42_** NC—(i) strong NIR absorption, (ii) a broad optical transparency window in the visible region, and (iii) efficient triplet generation and transfer—enabled the simultaneous achievement of record‐setting UC quantum yields (*Φ*_UC_) and significantly low threshold intensities (*I*_th_). Under 808 nm excitation, the system exhibited a *Φ*_UC_ (50% maximum) of 16.5% (reabsorption‐corrected *Φ*_UC_ of 21.4%) with an exceptionally low *I*_th_ of 0.14 W cm^−2^. Furthermore, under 936 nm excitation, a *Φ*_UC_ of 12.3% (reabsorption‐corrected *Φ*_UC_ of 15.0%) was attained, surpassing previously reported values above 850 nm by over two orders of magnitude. Quantitative analysis further revealed a high spin‐statistical factor (*f* = 0.58 ± 0.05) for rubrene, suggesting that *Φ*_UC_ values approaching 30% are theoretically possible with this annihilator. These remarkable performances originate from the distinctive electronic structure of **Au_42_**, which combines intense NIR absorption with high visible transparency, thereby effectively suppressing upconverted‐photon losses. These characteristics are expected to be shared by other compositionally diverse, needle‐shaped gold NCs [73, 74, 75, 76], underscoring their potential as a general class of NIR‐responsive triplet sensitizers. These features may pave the way for highly efficient, low‐threshold NIR‐to‐visible UC systems operating over a broad NIR wavelength range. This could open new avenues in solar‐energy conversion and advanced photonic applications.

## Conflicts of Interest

The authors declare no conflicts of interest.

## Supporting information



The authors have cited additional references within the Supporting Information [19, 24, 25, 26, 27, 28, 29, 30, 31, 32, 33, 34, 35, 36, 37, 38, 39, 40, 41, 42, 43, 44, 45, 46, 47, 48, 49, 50, 58, 60, 61, 64]. **Supporting File**: anie71420‐sup‐0001‐SuppMat.docx.

## Data Availability

The data that support the findings of this study are available in the supplementary material of this article.

## References

[1] P. Chen , Y. Xiao , S. Li , et al., “The Promise and Challenges of Inverted Perovskite Solar Cells,” Chemical Reviews 124 (2024): 10623–10700, 10.1021/acs.chemrev.4c00073. 39207782

[2] G. Zhang , F. R. Lin , F. Qi , et al., “Renewed Prospects for Organic Photovoltaics,” Chemical Reviews 122 (2022): 14180–14274, 10.1021/acs.chemrev.1c00955. 35929847

[3] Q. Wang and K. Domen , “Particulate Photocatalysts for Light‐Driven Water Splitting: Mechanisms, Challenges, and Design Strategies,” Chemical Reviews 120, no. 2 (2020): 919–985, 10.1021/acs.chemrev.9b00201. 31393702

[4] S. Balusche , T. Miteva , V. Yakutkin , G. Nelles , A. Yasuda , and G. Wegner , “Up‐Conversion Fluorescence: Noncoherent Excitation by Sunlight,” Physical Review Letters 97 (2006): 143903, 10.1103/PhysRevLett.97.143903. 17155253

[5] J. Zhou , Q. Liu , W. Feng , Y. Sun , and F. Li , “Upconversion Luminescent Materials: Advances and Applications,” Chemical Reviews 115 (2015): 395–465, 10.1021/cr400478f. 25492128

[6] T. N. Singh‐Rachford and F. N. Castellano , “Photon Upconversion Based on Sensitized Triplet–Triplet Annihilation,” Coordination Chemistry Reviews 254 (2010): 2560–2573, 10.1016/j.ccr.2010.01.003.

[7] A. Monguzzi , R. Tubino , S. Hoseinkhani , M. Campione , and F. Meinardi , “Low Power, Non‐Coherent Sensitized Photon Up‐conversion: Modelling and Perspectives,” Physical Chemistry Chemical Physics 14 (2012): 4322, 10.1039/c2cp23900k. 22370856

[8] V. Gray , D. Dzebo , M. Abrahamsson , B. Albinsson , and K. Moth‐Poulsen , “Triplet–Triplet Annihilation Photon‐Upconversion: Towards Solar Energy Applications,” Physical Chemistry Chemical Physics 16 (2014): 10345–10352, 10.1039/C4CP00744A. 24733519

[9] T. W. Schmidt and F. N. Castellano , “Photochemical Upconversion: The Primacy of Kinetics,” Journal of Physical Chemistry Letters 5 (2014): 4062–4072, 10.1021/jz501799m. 26276495

[10] N. Yanai and N. Kimizuka , “New Triplet Sensitization Routes for Photon Upconversion: Thermally Activated Delayed Fluorescence Molecules, Inorganic Nanocrystals, and Singlet‐to‐Triplet Absorption,” Accounts of Chemical Research 50 (2017): 2487–2495, 10.1021/acs.accounts.7b00235. 28930435

[11] L. Frazer , J. K. Gallaher , and T. W. Schmidt , “Optimizing the Efficiency of Solar Photon Upconversion,” ACS Energy Letters 2 (2017): 1346–1354, 10.1021/acsenergylett.7b00237.

[12] P. Bharmoria , H. Bildirir , and K. Moth‐Poulsen , “Triplet–triplet Annihilation Based Near Infrared to Visible Molecular Photon Upconversion,” Chemical Society Reviews 49 (2020): 6529–6554, 10.1039/D0CS00257G. 32955529

[13] A. J. Carrod and V. Gray , “Recent Advances in Triplet–Triplet Annihilation Upconversion and Singlet Fission, Towards Solar Energy Applications,” Energy & Environmental Science 15 (2022): 4982–5016, 10.1039/D2EE01600A.

[14] K. Chen , Q. Luan , T. Liu , B. Albinsson , and L. Hou , “Semiconductor Nanocrystals‐Based Triplet‐Triplet Annihilation Photon‐Upconversion: Mechanism, Materials and Applications,” Responsive Materials 3 (2025): e20240030, 10.1002/rpm.20240030.

[15] L. Naimovičius , P. Bharmoria , and K. Moth‐Poulsen , “Triplet–Triplet Annihilation Mediated Photon Upconversion Solar Energy Systems,” Materials Chemistry Frontiers 7 (2023): 2297–2315, 10.1039/D3QM00069A. 37313216 PMC10259159

[16] Y. Murakami and K. Kamada , “Kinetics of Photon Upconversion by Triplet–Triplet Annihilation: A Comprehensive Tutorial,” Physical Chemistry Chemical Physics 23 (2021): 18268–18282, 10.1039/D1CP02654B. 34612372

[17] F. Edhborg , A. Olesund , and B. Albinsson , “Best Practice in Determining Key Photophysical Parameters in Triplet–Triplet Annihilation Photon Upconversion,” Photochemical & Photobiological Sciences 21 (2022): 1143–1158, 10.1007/s43630-022-00219-x. 35441266

[18] S. Hoseinkhani , R. Tubino , F. Meinardi , and A. Monguzzi , “Achieving the Photon Up‐Conversion Thermodynamic Yield Upper Limit by Sensitized Triplet–Triplet Annihilation,” Physical Chemistry Chemical Physics 17 (2015): 4020–4024, 10.1039/C4CP03936J. 25574759

[19] N. Nishimura , V. Gray , J. R. Allardice , et al., “Photon Upconversion From Near‐Infrared to Blue Light With TIPS‐Anthracene as an Efficient Triplet–Triplet Annihilator,” ACS Materials Letters 1 (2019): 660–664, 10.1021/acsmaterialslett.9b00287.

[20] S. Mattiello , S. Mecca , A. Ronchi , et al., “Diffusion‐Free Intramolecular Triplet–Triplet Annihilation in Engineered Conjugated Chromophores for Sensitized Photon Upconversion,” ACS Energy Letters 7 (2022): 2435–2442, 10.1021/acsenergylett.2c01224.

[21] P. Baronas , J. Lekavičius , M. Majdecki , et al., “Automated Research Platform for Development of Triplet–Triplet Annihilation Photon Upconversion Systems,” ACS Central Science 11, no. 3 (2025): 413–421, 10.1021/acscentsci.4c02059. 40161950 PMC11950846

[22] D. Arima , S. Hidaka , S. Yokomori , et al., “Triplet‐Mediator Ligand‐Protected Metal Nanocluster Sensitizers for Photon Upconversion,” Journal of the American Chemical Society 146, no. 24 (2024): 16630–16638, 10.1021/jacs.4c03635. 38738855

[23] M. Mitsui , Y. Miura , R. Oyaizu , T. Yoshinami , and K. Kobayashi , “Photon Upconversion Enhanced by Ag_29_ Nanocluster Sensitizers and Multifunctional P(DPA)_3_ Acting as Triplet Mediator, Annihilator, and Emitter,” Journal of Physical Chemistry Letters 16 (2025): 10802–10810, 10.1021/acs.jpclett.5c02078. 41061749

[24] W. Liang , C. Nie , J. Du , et al., “Near‐Infrared Photon Upconversion and Solar Synthesis Using Lead‐Free Nanocrystals,” Nature Photonics 17 (2023): 346–353, 10.1038/s41566-023-01156-6.

[25] R. Sun , J. Zang , R. Lai , W. Yang , and B. Ji , “Near‐Infrared‐to‐Visible Photon Upconversion With Efficiency Exceeding 21% Sensitized by InAs Quantum Dots,” Journal of the American Chemical Society 146 (2024): 17618–17623, 10.1021/jacs.4c04997. 38899905

[26] V. Yakutkin , S. Aleshchenkov , S. Chernov , T. Miteva , G. Nelles , and A. Cheprakov , and S. Baluschev , “Towards the IR Limit of the Triplet–Triplet Annihilation‐Supported up‐Conversion: Tetraanthraporphyrin,” Chemistry—A European Journal 14 (2008): 9846–9850, 10.1002/chem.200801305. 18830987

[27] F. Deng , W. Sun , and F. N. Castellano , “Texaphyrin Sensitized Near‐IR‐to‐Visible Photon Upconversion,” Photochemical & Photobiological Sciences 13 (2014): 813–819, 10.1039/c4pp00037d. 24682185

[28] Z. Huang , X. Li , M. Mahboub , et al., “Hybrid Molecule–Nanocrystal Photon Upconversion Across the Visible and Near‐Infrared,” Nano Letters 15 (2015): 5552–5557, 10.1021/acs.nanolett.5b02130. 26161875

[29] S. Amemori , Y. Sasaki , N. Yanai , and N. Kimizuka , “Near‐Infrared‐to‐Visible Photon Upconversion Sensitized by a Metal Complex With Spin‐Forbidden yet Strong S_0_–T_1_ Absorption,” Journal of the American Chemical Society 138 (2016): 8702–8705, 10.1021/jacs.6b04692. 27354325

[30] M. Mahboub , Z. Huang , and M. L. Tang , “Efficient Infrared‐to‐Visible Upconversion With Subsolar Irradiance,” Nano Letters 16 (2016): 7169–7175, 10.1021/acs.nanolett.6b03503. 27788577

[31] Z. Huang , D. E. Simpson , M. Mahboub , X. Li , and M. L. Tang , “Ligand Enhanced Upconversion of near‐infrared Photons With Nanocrystal Light Absorbers,” Chemical Science 7 (2016): 4101–4104, 10.1039/C6SC00257A. 30155053 PMC6013917

[32] M. Mahboub , P. Xia , J. V. Baren , X. Li , C. H. Lui , and M. L. Tang , “Midgap States in PbS Quantum Dots Induced by Cd and Zn Enhance Photon Upconversion,” ACS Energy Letters 3 (2018): 767–772, 10.1021/acsenergylett.8b00122.

[33] Z. Huang , Z. Xu , M. Mahboub , et al., “Enhanced Near‐Infrared‐to‐Visible Upconversion by Synthetic Control of PbS Nanocrystal Triplet Photosensitizers,” Journal of the American Chemical Society 141 (2019): 9769–9772, 10.1021/jacs.9b03385. 31180212

[34] E. Radiunas , S. Raišys , S. Juršėnas , et al., “Understanding the Limitations of NIR‐to‐Visible Photon Upconversion in Phthalocyanine‐Sensitized Rubrene Systems,” Journal of Materials Chemistry C 8 (2020): 5525–5534, 10.1039/C9TC06031F.

[35] Y. Wei , K. An , X. Xu , Z. Ye , X. Yin , and X. Cao , “Π‐Radical Photosensitizer for Highly Efficient and Stable Near‐Infrared Photon Upconversion,” Advanced Optical Materials 12 (2024): 2301134, 10.1002/adom.202301134.

[36] E. Radiunas , L. Naimovičius , P. Baronas , A. Jozeliūnaitė , E. Orentas , and K. Kazlauskas , “CN‐Tuning: A Pathway to Suppress Singlet Fission and Amplify Triplet‐Triplet Annihilation Upconversion in Rubrene,” Advanced Optical Materials 13 (2025): 2403032, 10.1002/adom.202403032.

[37] L. H. Jiang , X. Miao , M. Y. Zhang , et al., “Near Infrared‐II Excited Triplet Fusion Upconversion With Anti‐Stokes Shift Approaching the Theoretical Limit,” Journal of the American Chemical Society 146 (2024): 10785–10797, 10.1021/jacs.4c00936. 38573588

[38] K. T. Chang , W. Liang , S. Gong , et al., “Triplet Sensitization Photon Upconversion Using Near‐Infrared Indirect‐Bandgap AgBiS_2_ Nanocrystals,” Journal of the American Chemical Society 147 (2025): 14015–14023, 10.1021/jacs.5c04015. 40204674 PMC12023030

[39] T. N. Singh‐Rachford , A. Nayak , M. L. Muro‐Small , S. Goeb , M. J. Therien , and F. N. Castellano , “Supermolecular‐Chromophore‐Sensitized Near‐Infrared‐to‐Visible Photon Upconversion,” Journal of the American Chemical Society 132 (2010): 14203–14211, 10.1021/ja105510k. 20828165

[40] S. Amemori , N. Yanai , and N. Kimizuka , “Metallonaphthalocyanines as Triplet Sensitizers for Near‐Infrared Photon Upconversion Beyond 850 Nm,” Physical Chemistry Chemical Physics 17 (2015): 22557–22560, 10.1039/C5CP02733K. 26270770

[41] B. D. Ravetz , A. B. Pun , E. M. Churchill , D. N. Congreve , T. Rovis , and L. M. Campos , “Photoredox Catalysis Using Infrared Light via Triplet Fusion Upconversion,” Nature 565 (2019): 343–346, 10.1038/s41586-018-0835-2. 30651612 PMC6338432

[42] N. Nishimura , J. R. Allardice , J. Xiao , Q. Gu , V. Gray , and A. Rao , “Photon Upconversion Utilizing Energy Beyond the Band Gap of Crystalline Silicon With a Hybrid TES‐ADT/PbS Quantum Dots System,” Chemical Science 10 (2019): 4750–4760, 10.1039/C9SC00821G. 31160951 PMC6510314

[43] E. M. Gholizadeh , S. K. K. Prasad , Z. L. Teh , et al., “Photochemical Upconversion of Near‐Infrared Light From Below the Silicon Bandgap,” Nature Photonics 14 (2020): 585–590, 10.1038/s41566-020-0664-3.

[44] L. Huang , W. Wu , Y. Li , et al., “Highly Effective Near‐Infrared Activating Triplet–Triplet Annihilation Upconversion for Photoredox Catalysis,” Journal of the American Chemical Society 142 (2020): 18460–18470, 10.1021/jacs.0c06976. 33074671

[45] A. T. Gilligan , R. Owens , E. G. Miller , N. F. Pompetti , and N. H. Damrauer , “Enhancing NIR‐to‐Visible Upconversion in a Rigidly Coupled Tetracene Dimer: Approaching Statistical Limits for Triplet–Triplet Annihilation Using Intramolecular Multiexciton States,” Chemical Science 15 (2024): 1283–1296, 10.1039/D3SC04795D. 38274080 PMC10806848

[46] Y. Sasaki , S. Amemori , H. Kouno , N. Yanai , and N. Kimizuka , “Near Infrared‐to‐Blue Photon Upconversion by Exploiting Direct S–T Absorption of a Molecular Sensitizer,” Journal of Materials Chemistry C 5 (2017): 5063–5067, 10.1039/C7TC00827A.

[47] K. Mase , Y. Sasaki , Y. Sagara , et al., “Stimuli‐Responsive Dual‐Color Photon Upconversion: A Singlet‐to‐Triplet Absorption Sensitizer in a Soft Luminescent Cyclophane,” Angewandte Chemie, International Edition 57 (2018): 2806–2810, 10.1002/anie.201712644. 29363244

[48] Y. Sasaki , M. Oshikawa , P. Bharmoria , et al., “Near‐Infrared Optogenetic Genome Engineering Based on Photon‐Upconversion Hydrogels,” Angewandte Chemie, International Edition 58 (2019): 17827–17833, 10.1002/anie.201911025. 31544993

[49] R. Haruki , Y. Sasaki , K. Masutani , N. Yanai , and N. Kimizuka , “Leaping Across the Visible Range: Near‐Infrared‐to‐Violet Photon Upconversion Employing a Silyl‐Substituted Anthracene,” Chemical Communications 56 (2020): 7017–7020, 10.1039/D0CC02240C. 32441716

[50] Y. Y. Cheng , B. Fückel , T. Khoury , et al., “Kinetic Analysis of Photochemical Upconversion by Triplet–Triplet Annihilation: Beyond any Spin Statistical Limit,” Journal of Physical Chemistry Letters 1 (2010): 1795–1799, 10.1021/jz100566u.

[51] Y. Y. Cheng , T. Khoury , R. G. C. R. Clady , et al., “On the Efficiency Limit of Triplet–Triplet Annihilation for Photochemical Upconversion,” Physical Chemistry Chemical Physics 12 (2010): 66–71, 10.1039/B913243K. 20024445

[52] A. Sawa , S. Shimada , N. Tripathi , et al., “Enhancing NIR‐to‐visible Photon Upconversion in Cast Solid by Introducing Bulky Substituents in Rubrene and by Suppressing Back Energy Transfer,” Journal of Materials Chemistry C 11 (2023): 8502–8513, 10.1039/D3TC00853C.

[53] D. G. Bossanyi , Y. Sasaki , S. Wang , et al., “Spin Statistics for Triplet–Triplet Annihilation Upconversion: Exchange Coupling, Intermolecular Orientation, and Reverse Intersystem Crossing,” JACS Au 1 (2021): 2188–2201, 10.1021/jacsau.1c00322. 34977890 PMC8715495

[54] M. Mitsui , “Recent Advances in Understanding Triplet States in Metal Nanoclusters: Their Formation, Energy Transfer, and Applications in Photon Upconversion,” Journal of Physical Chemistry Letters 15 (2024): 12257–12268, 10.1021/acs.jpclett.4c03003. 39636297

[55] Y. Niihori and M. Mitsui , “Harnessing Metal Cluster Sensitizers for Triplet–Triplet Annihilation Photon Upconversion: Strategies for Performance Enhancement,” Chemical Physics Review 6 (2025): 031301, 10.1063/5.0273181.

[56] S. Takano , H. Hirai , T. Nakashima , T. Iwasa , T. Taketsugu , and T. Tsukuda , “Photoluminescence of Doped Superatoms M@Au_12_ (M = Ru, Rh, Ir) Homoleptically Capped by (Ph_2_)PCH_2_P(Ph_2_): Efficient Room‐Temperature Phosphorescence From Ru@Au_12_ ,” Journal of the American Chemical Society 143 (2021): 10560–10564, 10.1021/jacs.1c05019. 34232036

[57] Y. Niihori , Y. Wada , and M. Mitsui , “Single Platinum Atom Doping to Silver Clusters Enables NearInfrared‐to‐Blue Photon Upconversion,” Angewandte Chemie, International Edition 60 (2021): 2822–2827, 10.1002/anie.202013725. 33295118

[58] M. Mitsui , Y. Wada , R. Kishii , D. Arima , and Y. Niihori , “Evidence for Triplet‐State‐Dominated Luminescence in Biicosahedral Superatomic Molecular Au_25_ Clusters,” Nanoscale 14 (2022): 7974–7979, 10.1039/D2NR00813K. 35470826

[59] M. Mitsui , Y. Miyoshi , and D. Arima , “Tailoring Sensitization Properties and Improving Near‐Infrared Photon Upconversion Performance Through Alloying in Superatomic Molecular Au_25_ Nanoclusters,” Nanoscale 16 (2024): 14757–14765, 10.1039/D4NR01948B. 38973468

[60] Z. Liu , X. Hu , L. Luo , et al., “Near‐Infrared to Visible Photon Upconversion With Gold Quantum Rods and Aqueous Photo‐Driven Polymerization,” Journal of the American Chemical Society 147 (2025): 28241–28250, 10.1021/jacs.5c08826. 40717289 PMC12333327

[61] M. Mitsui , D. Arima , Y. Kobayashi , E. Lee , and Y. Niihori , “On the Origin of Photoluminescence Enhancement in Biicosahedral Ag_ *x* _Au_25–*x* _ Nanoclusters (*x* = 0–13) and Their Application to Triplet–Triplet Annihilation Photon Upconversion,” Advanced Optical Materials 10 (2022): 2200864, 10.1002/adom.202200864.

[62] D. Arima and M. Mitsui , “Structurally Flexible Au–Cu Alloy Nanoclusters Enabling Efficient Triplet Sensitization and Photon Upconversion,” Journal of the American Chemical Society 145 (2023): 6994–7004, 10.1021/jacs.3c00870. 36939572

[63] M. Mitsui and A. Uchida , “Triplet Properties and Intersystem Crossing Mechanism of PtAg_28_ Nanocluster Sensitizers Achieving Low Threshold and Efficient Photon Upconversion,” Nanoscale 16 (2024): 3053–3060, 10.1039/D3NR05992H. 38240331

[64] L. Zeng , W.‐Q. Shi , J. Kong , et al., “Triplet Energy Transfer and Photon Upconversion From Metal Nanocluster With Near‐Unity NIR Emission Quantum Yield,” Advanced Optical Materials 13 (2025): 2402991, 10.1002/adom.202402991.

[65] W. Zhang , T. Xu , J. Kong , et al., “Intensive Near‐Infrared Emitting Au_7_Cu_10_ Nanoclusters for Both Energy and Electron Harvesting,” Chemical Science 16 (2025): 8910–8921, 10.1039/D5SC00671F. 40271028 PMC12012834

[66] H. Hirai , S. Takano , T. Nakashima , T. Iwasa , T. Taketsugu , and T. Tsukuda , “Doping‐Mediated Energy‐Level Engineering of M@Au_12_ Superatoms (M = Pd, Pt, Rh, Ir) for Efficient Photoluminescence and Photocatalysis,” Angewandte Chemie, International Edition 61 (2022): e202207290, 10.1002/anie.202207290. 35608869

[67] L. Luo , Z. Liu , X. Du , and R. Jin , “Near‐Infrared Dual Emission From the Au_42_(SR)_32_ Nanocluster and Tailoring of Intersystem Crossing,” Journal of the American Chemical Society 144 (2022): 19243–19247, 10.1021/jacs.2c09107. 36239690

[68] P. Chandrasekar , G. Sardar , T. Sengupta , et al., “Modulation of Singlet‐Triplet Gap in Atomically Precise Silver Cluster‐Assembled Material,” Angewandte Chemie, International Edition 63 (2024): e202317345, 10.1002/anie.202317345. 38078805

[69] N. L. Smith and K. L. Knappenberger Jr. , “Influence of Aliphatic Versus Aromatic Ligand Passivation on Intersystem Crossing in Au_25_(SR)_18_ ^–^ ,” Journal of Physical Chemistry A 128 (2024): 7620–7627, 10.1021/acs.jpca.4c04387. 39197122

[70] K. Li , P. Wang , and Y. Pei , “Impact of the Peripheral Ligand Layer on the Excited‐State Deactivation Mechanism of Au_38_S_2_ (S‐Adm)_20_ and Au_30_ (S‐Adm)_18_ (S‐Adm = Adamantanethiolate) Clusters,” Journal of Physical Chemistry Letters 15 (2024): 9216–9225, 10.1021/acs.jpclett.4c02246. 39225489

[71] A. Mazumder , K. Li , Z. Liu , et al., “Isomeric Effects of Au_28_ (S‐c‐C_6_H_11_)_20_ Nanoclusters on Photoluminescence: Roles of Electron‐Vibration Coupling and Higher Triplet State,” ACS Nano 18 (2024): 21534–21543, 10.1021/acsnano.4c06702. 39092525 PMC11328167

[72] L. Zeng , Y. Wang , J. Tan , et al., “Accelerated Intersystem Crossing Enhances NIR Emission in Au_52_(SR)_32_ Nanoclusters by Surface Ligand Engineering,” Chemical Science 16 (2025): 18844–18851, 10.1039/D5SC03898G. 40963548 PMC12438965

[73] L. Luo , Z. Liu , J. Kong , et al., “Three‐Atom‐Wide Gold Quantum Rods With Periodic Elongation and Strongly Polarized Excitons,” Proceedings National Academy of Science USA 121 (2024): e2318537121, 10.1073/pnas.2318537121. PMC10927531 38412123

[74] Y. Li , Y. Song , X. Zhang , et al., “Atomically Precise Au_42_ Nanorods With Longitudinal Excitons for an Intense Photothermal Effect,” Journal of the American Chemical Society 144 (2022): 12381–12389, 10.1021/jacs.2c03948. 35767839

[75] S. Takano , Y. Hamasaki , and T. Tsukuda , “X‐Ray Crystallographic Visualization of a Nucleation and Anisotropic Growth in Thiolate‐Protected Gold Clusters: Toward Targeted Synthesis of Gold Quantum Needles,” Journal of the American Chemical Society 147, no. 37 (2025): 33953–33962, 10.1021/jacs.5c11089. 40908517 PMC12447497

[76] Y. Hamasaki , R. Jonoue , S. Takano , and T. Tsukuda , “Gold Quantum Needles Synthesized by Thermal‐Induced, One‐Dimensional Oligomerization of Au_24_(SC_2_H_4_Ph)_20_ ,” Journal of the American Chemical Society 147, no. 49 (2025): 44680–44685, 10.1021/jacs.5c14201. 41287142 PMC12703669

[77] X.‐Y. Xie , K.‐Q. Cheng , W.‐K. Chen , et al., “Near‐Infrared Dual‐Emission of a Thiolate‐Protected Au_42_ Nanocluster: Excited States, Nonradiative Rates, and Mechanism,” Journal of Physical Chemistry Letters 14 (2023): 10025–10031, 10.1021/acs.jpclett.3c02683. 37906639

[78] Y. Luo , K. Li , P. Wang , and Y. Pei , “Structural Flexibility‐Driven Dual Emission Switching in Ultrathin Gold Nanorod Clusters,” JACS Au 5 (2025): 4593–4603, 10.1021/jacsau.5c00898. 41001636 PMC12458031

[79] G. Orellana and A. Braun , “Quantum Yields of ^3^MLCT Excited state Formation and Triplet—Triplet Absorption Spectra of Ruthenium(II) Tris‐chelate Complexes Containing Five‐ and Six‐Membered Heterocyclic Moieties,” Journal of Photochemistry and Photobiology A: Chemistry 48 (1989): 277–289, 10.1016/1010-6030(89)87009-1.

[80] K. Sandros and H. L. J. Backstrom , “Transfer of Triplet State Energy in Fluid Solutions. II. Further Studies of the Quenching of Biacetyl Phosphorescence in Solution,” Acta Chemica Scandinavica 16 (1962): 958–968, 10.3891/acta.chem.scand.16-0958.

[81] Y. Li , Y. Song , X. Zhang , et al., “Atomically Precise Au_42_ Nanorods With Longitudinal Excitons for an Intense Photothermal Effect,” Journal of the American Chemical Society 144 (2022): 12381–12389, 10.1021/jacs.2c03948. 35767839

[82] J. T. Dubose , G. Szabó , J. Chakkamalayath , and P. V. Kamat , “Excited‐State Transient Chemistry of Rubrene: A Whole Story,” Journal of Physical Chemistry A 126 (2022): 7147–7158, 10.1021/acs.jpca.2c04499. 36074750

[83] N. Kiseleva , D. Busko , B. S. Richards , M. A. Filatov , and A. Turshatov , “Determination of Upconversion Quantum Yields Using Charge‐Transfer State Fluorescence of Heavy‐Atom‐Free Sensitizer as a Self‐Reference,” Journal of Physical Chemistry Letters 11 (2020): 6560–6566, 10.1021/acs.jpclett.0c01902. 32702988

[84] K. Kamada , Y. Sakagami , T. Mizokuro , et al., “Efficient Triplet–Triplet Annihilation Upconversion in Binary Crystalline Solids Fabricated via Solution Casting and Operated in Air,” Materials Horizons 4 (2017): 83–87, 10.1039/C6MH00413J.

[85] M. Mitsui , Y. Ohshima , and O. Kajimoto , “Structure and Dynamics of 9(10H)‐Acridone and Its Hydrated Clusters. III. Microscopic Solvation Effects on Nonradiative Dynamics,” Journal of Physical Chemistry A 104 (2000): 8660–8670, 10.1021/jp001049u.

[86] J. H. Baek , D. Song , G. Park , et al., “Highly Efficient Upconverted Fluorescence From Coulombically Assembled Ion Pair,” Advanced Optical Materials 13 (2025): 2500876, 10.1002/adom.202500876.

[87] M. Montalti , A. Credi , L. Prodi , and M. T. Gandolfi , Handbook of Photochemistry. 3rd ed. (CRC Press, 2006), 10.1201/9781420015195.

[88] I. Moreels , K. Lambert , D. Smeets , et al., “Size‐Dependent Optical Properties of Colloidal PbS Quantum Dots,” ACS Nano 3, no. 10 (2009): 3023–3030, 10.1021/nn900863a. 19780530

[89] P. Baronas , J. Lekavičius , M. Majdecki , et al., “Automated Research Platform for Development of Triplet–Triplet Annihilation Photon Upconversion Systems,” ACS Central Science 11, no. 3 (2025): 413–421, 10.1021/acscentsci.4c02059. 40161950 PMC11950846

