# Supplementary material for: Au42(PET)32 Nanocluster Sensitizer Unlocks the Annihilator Potential of Rubrene, Enabling High‐Performance NIR‐to‐Visible Photon Upconversion
Source: Angew Chem Int Ed Engl. 2026 Feb 12;65(13):e23868. doi: 10.1002/anie.202523868 (PMC13007585; doi:10.1002/anie.202523868)
Supplement: Supplementary file 1 — The authors have cited additional references within the Supporting Information [19, 24, 25, 26, 27, 28, 29, 30, 31, 32, 33, 34, 35, 36, 37, 38, 39, 40, 41, 42, 43, 44, 45, 46, 47, 48, 49, 50, 58, 60, 61, 64]. Supporting File: anie71420‐sup‐0001‐SuppMat.docx. [file ANIE-65-e23868-s001.docx]

Supporting Information

Au_42_(PET)_32_ Nanocluster Sensitizer Unlocks the Annihilator Potential of Rubrene, Enabling High-Performance Near-Infrared-to-Visible Photon Upconversion

**Masaaki Mitsui,^†*^ Shinjiro Takano,^‡*^ Tatsuya Tsukuda^‡*^**

†Department of Chemistry, College of Science, Rikkyo University, 3-34-1,

Nishiikebukuro, Toshima-ku, Tokyo 171-8501, Japan

‡Department of Chemistry, Graduate School of Science, The University of Tokyo,

7-3-1, Hongo, Bukyo-ku, Tokyo 113-0033, Japan

Experimental Procedures

**Chemicals**

Solvents except for dichloromethane (DCM) and tetrahydrofuran (THF), and triethylamine (NEt_3_) were purchased from Fujifilm Wako Pure Chemical Industries. DCM (dehydrated) and THF (dehydrated and stabilizer-free) were purchased from Kanto Chemicals. 2-phenylethanethiol (PET-H) and cesium acetate (CsOAc) were purchased from Tokyo Chemical Industry. Toluene for spectroscopic measurements, rubrene (>99%, purified by sublimation), *N*,*N*’-bis(2,5-di-*tert*-butylphenyl)-3,4,9,10-perylenedicarboximide (*^t^*Bu-PDI), tetrabutylammonium hexafluorophosphate (TBAPF_6_) and *tert*-butylamine-borane (TBAB) complex was purchased from Sigma-Aldrich. Preparative thin layer chromatography (PTLC) plate was purchased from Miles Scientific (UNIPLATES; P02013). The water used was Milli-Q grade (>18 MΩ). All commercially available reagents were used as received except for TBAPF_6_, which was recrystallized from EtOH and vacuum dried for a few days at 80 °C.

**General**

Positive-mode ESI-mass spectra were recorded on a Bruker compact time-of-flight mass spectrometer. A toluene solution of samples was mixed with 10 mM ethanolic solution of CsOAc (v:v = 1:1) and centrifuged at 12000 rpm for 2 min. The supernatant were directly infused at a flow rate of 5 µL/min via a syringe pump. The measurement parameters were as follows: spray voltage 5 kV; end plate offset: 200 V; nebulizer: 0.3 bar; dry gas: 4 L/min; dry temp.: 120°C; isCID energy: 100 eV; ion energy: 4 eV; collision energy: 5 eV; transfer time: 350 µs; and pre pulse storage: 32 µs. The spectra were calibrated using Cs^+^(CsOAc)*_n_* clusters as an internal standard. The isotope patterns were calculated using mMass software.^1^ Elemental analysis was carried out at the Microanalytical Laboratory, Graduate School of Science, The University of Tokyo. Electrochemical measurement was conducted using EC FRONTIER model ECstat-400 potentiostat equipped with a Fraday cage. The **Au_42_** sample was dissolved in 0.1 M DCM solution of TBAPF_6_ as a supporting electrolyte with a final sample concentration of ca. 0.5 mM. The electrochemical cell consisted of a Pt wire counter electrode, and Ag wire reference electrode which was soaked in the supporting electrolyte solution separated by ion permeable glass, a glassy carbon working electrode (φ1 mm), an Ar gas inlet, and a gas outlet. The working electrode was polished using 0.05 µm Al_2_O_3_ paste and the polished electrode was successively sonicated with water and EtOH. After sonication, the polished electrode was rinsed with acetone and dried by a stream of Ar. Differential pulse voltammogram (DPV) was recorded using the following pulse program: potential increment: 4 mV; pulse height: 50 mV; pulse width: 50 ms; pulse period: 200 ms; 1st sampling timing: 100–120 ms; 2nd sampling timing: 180-200 ms. After the measurement, ferrocene was added to the sample solution and the measured potential was calibrated to the ferrocenium/ferrocene redox couple.

**Synthesis of Au_42_(PET)_32_**

The Au_42_(PET)_32_ nanocluster (**Au_42_**) was synthesized in the following two steps without the use of *N*-heterocycliccarbene salt reported by Jin.^2^

**First step:** A 200 mL round-bottom flask was charged with 100 mL of THF and a magnetic stir bar. While stirring, the AuClSMe_2_ complex (354 mg, 1.2 mmol, 1 eq.) was added to the flask, forming a slightly turbid solution. Immediately after the forming the turbid solution (<2 min.), neat PET-H (128.6 µL, 0.96 mmol, 4/5 eq.) was added dropwise to the Au solution. This resulted in a pale yellow, clear solution (solution A). Solution A was stirred for a minimum of 5 min. at room temperature. Meanwhile, a 30 mL vial was charged with solid TBAB (17.4 mg, 0.2 mmol, 1/6 eq.), which was dissolved by adding 20 mL of DCM and NEt_3_ (167 µL, 1.2 mmol, 1 eq.) (solution B). Then, solution B was added dropwise to the solution A over 5 min. During this addition, white fumes formed (presumably NEt_3_∙HCl), and the solution was gradually turned to turbid yellow at first, then orange, and finally reddish black. After stirring for 3 h, the solution was evaporated to dryness, and the solid was thoroughly washed with MeOH. The dried solid was extracted with 20 mL of DCM, and the extract was evaporated to dryness leaving a black solid (~380 mg).

**Second step:** The crude product obtained was dissolved in 1,2-dichloroethane (DCE) at a concentration of ca. 100 mg/mL in a test tube with a lid. The DCE solution was heated at 65 °C for 40 h to eliminate meta-stable species. The mixture was then centrifuged to remove the insoluble solid, and the resulting supernatant was evaporated to dryness, yielding a black solid (~300 mg). This solid was then extracted with DCM, and the mixture was purified using preparative thin-layer chromatography (SiO_2_; DCM:*n-*Hex = 1:1). The separated bands were cut off and extracted with DCM. From the greenish band with Rf~0.25, we collected 44 mg of **Au_42_** (12 % yield based on Au). Although the obtained samples exhibited the characteristic absorption profiles of **Au_42_** (Figure S2a), further purification was conducted using a GPC to guarantee the purity of the products for quantitative analysis (35 mg) (Figure S2b and c). Analytical data for **Au_42_**: UV-vis-NIR (toluene, λ(nm)): 807, 660(hump), 571(hump), 440(shoulder), 378; ESI-MS (positive, most abundant): 6464.3685 (observed as [M+2Cs]^2+^), 6464.3799 (calcd.); Elemental analysis calcd. for Au_42_(SC_8_H_9_)_32_: C 24.28, H 2.29, N 0.00, S 8.10; found: C 24.30, H 2.10, N 0.00, S 8.43.

**Theoretical calculations**

Density functional theory (DFT) and time-dependent DFT (TD-DFT) calculations were performed using the *Gaussian 16* program package (ES64L-G16, Rev. B.01).^3^ The ground-state geometry of Au_42_(PET)_32_ was optimized at the PBE0 level of theory^4^ with the def2-SV(P) basis set^5^ for Au atoms and the 6-31G(d) basis set^6^ for H, C, and S atoms. Scalar relativistic effects were included via the SDD (Stuttgart/Dresden) pseudopotentials.^7^ No symmetry constraints were imposed during the optimization. To confirm that the optimized structure corresponded to a true local minimum, harmonic vibrational frequency analysis was carried out to ensure the absence of imaginary frequencies. Based on the optimized structure, vertical excitation energies for S₀ → S*ₙ* (*n* = 1–200) transitions were computed using the TD-CAM-B3LYP functional.^8^ The same combination of basis sets and pseudopotentials was employed in these TD-DFT calculations. For the aromatic molecules used in the quenching experiments, geometrical optimizations and harmonic vibrational frequency analyses were performed at the M06-2X/6-31G(d,p) level of theory.^9^ Geometries and molecular orbitals were visualized using *GaussView 6.0* and *Avogadro 1.2.0*.

**Absorption and emission measurements**

Ultraviolet–visible absorption spectra were recorded using either a Lambda 650 spectrometer (PerkinElmer) or a V-770 spectrophotometer (JASCO) over the wavelength range of 300–2,500 nm with 1 nm resolution. Near-infrared (NIR) emission spectra were obtained by splitting the emission signal from the sample solution via a bifurcated optical fiber (QBIF400-MIXED, Ocean Insight) into a visible-range spectrometer (Ocean SR6, Ocean Insight) and a NIR spectrometer (NIRQuest+1.7, Ocean Insight). The collected spectra were merged using OceanView 2.0 software (Ocean Insight), enabling continuous spectral detection over the 500–1700 nm range. The spectral sensitivity of both spectrometers was calibrated using a standard light source (HL-3P-INT-CAL, Ocean Photonics) under optical conditions identical to those employed during the measurements.

**Absolute quantum yield and time-resolved emission measurements**

Absolute emission quantum yield measurements were performed using Hamamatsu Photonics C9920-02G absolute emission quantum yield measurement system. Details of the experimental setup for time-resolved emission measurements have been described in our previous reports.^10–13^ Time-resolved experiments were conducted using either a home-built time-correlated single-photon counting (TC-SPC) system or the TC-SPC mode of a PicoTAS setup (UNISOKU Co., Ltd.). In the custom-built system, excitation was provided by a 634 nm picosecond pulsed laser (pulse width: 40 ps; PiL063X, Advanced Laser Diode Systems), and emission decays were detected using an avalanche photodiode (APD, SPCM-AQRH-61, PerkinElmer) coupled with a TC-SPC module (TimeHarp 260, PicoQuant). Data analysis was carried out using SymPhoTime 64 software (PicoQuant). In the PicoTAS setup, excitation was achieved using a supercontinuum source (INDUS FORTE 400, Leukos; 20 MHz repetition rate; pulse width = 50–100 ps; 410–2400 nm spectral range). All emission measurements were conducted in toluene solutions thoroughly degassed with high-purity argon gas (>99.999%).

**Transient absorption measurements**

Transient absorption (TA) spectroscopy was performed using a subnanosecond TA system (picoTAS, UNISOKU Co., Ltd.).^14^ Excitation was provided by a picosecond Nd:YAG laser (355 nm, EKSPLA PL-2210A, 1 kHz, full width at half maximum (fwhm) = 25 ps), combined with an optical parametric generator (EKSPLA PT400, 410–700 nm, 50 μJ/pulse@500 nm). A supercontinuum light source (INDUS FORTE 400, Leukos; 20 MHz; fwhm = 50–100 ps; 410–2400 nm) served as the probe. The system provided a time resolution of approximately 80–100 ps (10–90% rise time). All TA measurements were conducted in a 2 mm path length quartz cuvette. For the **Au_42_** (6 μM)/rubrene (15 mM) mixture, spectral measurements below 570 nm were hampered by strong absorption of the probe light by ground-state rubrene at this high concentration.

**Evaluation of ISC quantum yield**

The intersystem crossing (ISC) quantum yield (Φ_ISC_) of **Au_42_** was determined from TA data using a partial-saturation method.^15^ Specifically, the dependence of the excited-state absorption (ESA) signal originating from the triplet (T₁) state of **Au_42_** in toluene on the 355 nm pump-pulse energy was measured at a delay time of 0.15 μs after excitation. The analysis is based on a two-state kinetic model consisting of the ground (S_0_) and triplet (T_1_) states, which yields the following relationships:

$$\text{Δ}\text{A}\left( \text{λ} \right)\text{ = }\text{a}\left\{ \text{1}\text{‒}\text{exp}\left( \text{‒}\text{b}\text{E}_{\text{ex}} \right) \right\}\text{, } \text{(S1)}$$

$$\text{a}\text{ = (}\text{ε}_{\text{T}}^{\text{*}}\text{ - }\text{ε}_{\text{0}}\text{) }\text{c}_{\text{0}}\text{l }\text{, } \text{(S2)}$$

$$\text{b}\text{ }\text{=}\text{ }\text{2303}\text{∙}\text{ε}_{\text{ex}}^{\text{0}}\text{Φ}_{\text{ISC}}, \text{(S3)}$$

where *E_ex_* is the excitation photon fluence (Einstein cm^-2^), $\text{ε}_{\text{T}}^{\text{*}}$ and $\text{ε}_{\text{0}}$ are the molar absorption coefficients of the triplet-excited and ground states, respectively, at the monitor wavelength (i.e., 1010 nm), *c*_0_ is the ground-state concentration prior to excitation, *l* is the optical path length, and $\text{ε}_{\text{ex}}^{\text{0}}$ is the molar absorption coefficient of the ground-state at the excitation wavelength. Fitting the experimental plot of Δ*A* versus *E_ex_* provides the parameters *a* and *b*, from which Φ_ISC_ and $\text{ε}_{\text{T}}^{\text{*}}$ were calculated. It should be noted that in Figure 2d, Δ*A*(1010) values are plotted as a function of the energy per pump pulse (J pulse⁻¹), and all calculations were performed with appropriate unit conversions.

**Global target analysis of transient absorption spectra**

The transient absorption data were analyzed by global target analysis using a sequential kinetic model.^16^ This approach allowed us to extract the species-associated spectra (SAS) and determine the triplet energy transfer (TET) rate constants (*k*_TET_) and efficiency. The data matrix *D*(*λ*,*t*), representing the change in absorbance as a function of wavelength (*λ*) and time (*t*), was decomposed into a concentration matrix *C*(*t*) and a matrix of SAS (SAS(*λ*)) according to the following equation:

$$\text{D}\text{(}\text{λ}\text{, t}\text{)}\text{ = }\text{C}\text{(}\text{t}\text{)}\text{∙SAS(}\text{λ}\text{)}\text{ }\text{+}\text{ }\text{E}\text{, }\text{(S}\text{4}\text{)}$$

where *E* represents the residual noise matrix. The concentration matrix *C*(*t*) was constructed based on a sequential kinetic model describing the donor-acceptor system:

The time-dependent concentrations were determined by solving the corresponding rate equations:

$$\left[ \text{D}^{\text{*}} \right]\left( \text{t} \right)\text{ = }\left[ \text{D}^{\text{*}} \right]_{\text{0}}\text{exp}\left[ \text{‒}\left( \frac{1}{\tau_{\text{D0}}}+\text{k}_{\text{TET}}\text{[A]} \right)\text{t} \right]\text{, } \text{(S5)}$$

$$\left[ \text{A}^{\text{*}} \right]\left( \text{t} \right)\text{ = }\frac{1/{\text{τ}_{\text{D0}}}+\text{k}_{\text{TET}}\text{[A]}}{1/{\tau_{\text{D0}}}+\text{k}_{\text{TET}}\text{[A]}-1/{\tau_{\text{A}}}}\left[ \text{D}^{\text{*}} \right]_{\text{0}}\text{exp}\left( \text{‒}\frac{1}{\tau_{\text{A}}}\text{t} \right)-\text{exp}\left[ \text{‒}\left( \frac{1}{\tau_{\text{D0}}}+\text{k}_{\text{TET}}\text{[A]} \right)\text{t} \right]\text{,} \text{(S6)}$$

where [D*]_0_ is the initial concentration of the donor in the excited triplet state; $\tau_{\text{D}\text{0}}$ is the triplet lifetime of the donor in the absence of the acceptor; $\text{k}_{\text{TET}}$ represents the second-order rate constant for TET; [A] is the concentration of the acceptor; and $\tau_{\text{A}}$ is the triplet state lifetime of the acceptor produced via the energy transfer process. An additional constant offset component was included to account for long-lived species or baseline shifts. The optimization was carried out using the lmfit library in Python by minimizing the weighted residuals. To account for the signal-to-noise ratio, the weighting factor (*w*) was defined as

$$w\text{ = }\text{1}/\left( \sqrt{\left| \text{D}_{\text{exp}} \right|}\text{+0.01∙max}\left| \text{D}_{\text{exp}} \right| \right)\text{ } \text{(S7)}$$

The SAS were extracted via a linear least-squares method at each iteration of the non-linear kinetic parameter optimization.

**Estimation of Gibbs free energy change of charge transfer**

Free energy changes (Δ*G*_CT_) of charge transfer (CT) between donor (D) and acceptors (A) can be calculated by the Rehm−Weller equation:

$$\text{∆}\text{G}_{\text{CT}}\text{ = }\text{F}\left[ \text{E}_{\text{D}}^{\text{ox}}\text{ ‒ }\text{E}_{\text{A}}^{\text{red}} \right]\text{ ‒ }\text{E}_{\text{ex}}\text{ + }\text{C}\text{,} \text{(S8)}$$

where *F* is the Faraday constant, $\text{E}_{\text{D}}^{\text{ox}}$ and $\text{E}_{\text{A}}^{\text{red}}$ are the oxidation and reduction potentials of the donor and acceptor, respectively, *E*_ex_ is the excited-state energy of **Au_42_**, and *C* is the coulombic interaction term between the charge-separated species. The coulombic energy can be estimated by following equation:^17^

$$\text{C}\text{ = }\frac{\text{z}_{\text{D}}\text{z}_{\text{A}}\text{e}^{\text{2}}}{\text{ε}_{\text{s}}\text{(}\text{R}_{\text{D}}\text{+}\text{R}_{\text{A}}\text{)}}\text{‒}\frac{\text{e}^{\text{2}}}{\text{2}}\text{(}\frac{\text{z}_{\text{D}}^{\text{2}}}{\text{R}_{\text{D}}}\text{+}\frac{\text{z}_{\text{A}}^{\text{2}}}{\text{R}_{\text{A}}}\text{)(}\frac{\text{1}}{\text{ε}_{\text{ref}}}\text{+}\frac{\text{1}}{\text{ε}_{\text{s}}}\text{),}\text{ } \text{(S}\text{9}\text{)}$$

where *z* represents the charge on donor and acceptor in the CT complex, *e* is the elementary charge, *ε*_s_ and *ε*_ref_ are the dielectric constants of the solvent used in the absorption/emission measurements and used in electrochemical measurements, respectively. *R* is the van der Waals radii of donor or acceptor. The parameters used and the calculated Δ*G*_CT_ values are summarized in Table S2.

**Dynamic light scattering measurements**

Dynamic light scattering (DLS) measurements were performed using a nanoSAQLA instrument (Otsuka Electronics Co., Ltd.). All measurements were conducted at 25 °C with a 660 nm laser as the light source. Particle size distributions were evaluated by analyzing the intensity autocorrelation function, *G^(^*^2)^(*t*), of the scattered light, using the dedicated nanoSAQLA software, with appropriate solvent parameters (e.g., viscosity and refractive index) as inputs. Measurements were carried out under the highest concentration conditions used for UC experiments, namely **Au_42_** (16 µM) and **Au_42_** (16 µM)/rubrene (20 mM) solutions.

**Upconversion emission decay measurement and analysis**

The experimental setup for UC emission decay measurements has been described in our previous reports.^10–13^ Briefly, a pseudo-pulsed laser excitation was generated by modulating a continuous-wave laser beam with an optical chopper operating at 1000 Hz. The UC emission signal was detected using an avalanche photodiode (APD410A/M, Thorlabs) and recorded with a digital oscilloscope (TBS1052C, Tektronix). Each decay profile was integrated over 300 cycles using custom LabVIEW 2020 software.

The temporal profile of the UC emission intensity, *I*_UC_(*t*), was analyzed using the following kinetic model:^18^

$$\text{I}_{\text{UC}}\left( \text{t} \right)\text{ }\text{∝}\text{ }\left[ {}_{\text{ }}^{\text{3}}\text{rubrene}\text{*} \right]_{\text{t}}^{\text{2}}\text{= }\text{I}\text{(0)}\left( \frac{\text{1}-\text{β}}{\exp\left( \frac{\text{t}}{\text{τ}_{\text{T}}} \right)-\text{β}} \right)^{\text{2}}\text{,}\text{ } \text{ (S}\text{10}\text{)}$$

where *I*(0) is the UC emission intensity at *t* = 0, *τ*_T_ is the decay time of the rubrene triplet state (³rubrene*) in the absence of TTA, and *β* is a dimensionless parameter representing the initial fraction of the TTA decay rate relative to the total triplet decay rate.

$$\text{β}\text{ = }\frac{\text{2}\text{k}_{\text{TTA}}{\text{[}{}_{\text{ }}^{\text{3}}\text{rubrene}\text{*]}}_{\text{0}}}{\text{τ}_{\text{T}}\text{ + 2}\text{k}_{\text{TTA}}{\text{[}{}_{\text{ }}^{\text{3}}\text{rubrene}\text{*]}}_{\text{0}}}\text{. }\text{ }\text{ (S}\text{11}\text{)}$$

**Evaluation of emission quantum yield**

The emission quantum yields (Φ_em_) were determined using the following equation:

$$\text{Φ}_{\text{em}}\text{= }\frac{\text{I}_{\text{r}}\left( \text{1}-\text{10}^{-\text{A}_{\text{r}}\left( \text{λ}_{\text{ex}} \right)} \right)}{\text{I}_{\text{o}}\left( \text{1}-\text{10}^{-\text{A}_{\text{o}}\left( \text{λ}_{\text{ex}} \right)} \right)}\frac{\int\text{F}_{\text{o}}\left( \text{λ}_{\text{em}} \right)\text{d}\text{λ}_{\text{em}}}{\int\text{F}_{\text{r}}\left( \text{λ}_{\text{em}} \right)\text{d}\text{λ}_{\text{em}}}\frac{{\text{n}_{\text{o}}}^{\text{2}}}{{\text{n}_{\text{r}}}^{\text{2}}}\text{Φ}_{\text{FL}}\text{,}\text{ (S}\text{12}\text{)}$$

where subscripts “r” and “o” denote the reference and objective samples, Φ_FL_ is the fluorescence quantum yield of the reference sample, *I* is the excitation light intensity used in the measurement, and *n* is the refractive index. *A*(*λ*_ex_) and *F*(*λ*_em_) represent the absorbance at the excitation wavelength, *λ*_ex_, and the intensity at the emission wavelength, *λ*_em_, respectively. The fluorescence (Φ_FL_) and phosphorescence (Φ_PH_) quantum yields of **Au_42_**, as well as the upconversion quantum yield (Φ_UC_), were evaluated under continuous-wave laser excitation at 808 ± 2 nm or 936 ± 9 nm using a laser diode (CivilLaser). For Φ_FL_, a deaerated toluene solution of *t*Bu-PDI (Φ_FL_ = 0.97)^19^ was employed as a reference standard and excited at 532 nm. In evaluating Φ_UC_, the fluorescence and phosphorescence components were deconvoluted on the energy axis, and the fluorescence band area was calculated after reconversion to the wavelength axis.^20^ The excitation beam area, determined by the knife-edge method, were 0.025 cm^2^ (808 nm), 0.036 cm^2^ (936 nm), and 0.023 cm^2^ (532 nm), respectively.

In the UC quantum yield measurements, an *edge-excitation* geometry was employed, wherein the region near the inner surface of the cuvette was selectively irradiated (Figure S10). Under these conditions, upconverted photons generated within the excitation zone traversed the shortest optical path through the solution, thereby markedly reducing reabsorption losses compared to a center-excitation configuration. Nevertheless, because of the extremely high annihilator concentration in the UC solution, complete suppression of reabsorption could not be achieved. To account for this effect, a widely accepted correction procedure was adopted: the observed UC spectrum was scaled so that its long-wavelength region overlapped that of a dilute rubrene solution, and the ratio of the integrated spectral areas was then used as the correction factor (Φ_out_) to obtain the internal quantum yield (Φ_UCg_).^21^

**Upconversion measurements under 1-sun irradiation**

The details of the experimental configuration were already described in elsewhere.^12^ Briefly, it employed for measuring upconverted emissions under the simulated AM1.5G light irradiation, generated by a solar simulator (XES-40S2-CE, San-ei Electric Manufacturing Co., Ltd.). The simulated AM1.5G light intensity was adjusted to 100 mWcm^−2^ by using a pyranometer (ML-01, EKO instruments). Appropriate optical filters were used to selectively irradiate the sample solution at wavelengths that do not directly excite the rubrene annihilator molecules. The UC emission spectra were obtained by detecting the emission signal with a visible-range spectrometer (Ocean SR6, Ocean Insight).

**FRET analysis**

According to Förster resonance energy transfer (FRET) theory,^20^ the FRET rate constant (*k*_FRET_) from a donor (D) to an acceptor (A) separated by a center-to-center distance *r* is given by

$$\text{k}_{\text{FRET}}\text{ = }\frac{\text{1}}{\text{τ}_{\text{D}}^{\text{0}}}\left[ \frac{\text{R}_{\text{0}}}{\text{r}} \right]^{\text{6}}\text{,}\text{ } \text{(S}\text{13}\text{)}$$

where $\tau_{D}^{0}$ is the S_1_-state lifetime of the donor in the absence of FRET, and *R*₀ is the Förster radius corresponding to 50% FRET efficiency (Φ_FRET_). The Förster radius is expressed as

$$\text{R}_{\text{0}}\text{ (in nm) =}{\text{ 0.02108}\left( \frac{\text{κ}^{\text{2}}\text{Φ}_{\text{D}}}{\text{n}^{\text{4}}}\text{J} \right)}^{\frac{\text{1}}{\text{6}}}\text{,}\text{ } \text{(S1}\text{4}\text{)}$$

where *κ*^2^ is the orientation factor, Φ_D_ is the donor FL quantum yield in the absence of acceptor, and *n* is the refractive index of the medium. In Equation S14, *J* (in M^−1^cm^−1^nm^4^) is the spectral overlap integral between the area-normalized FL spectrum of the donor and the absorption spectrum of the acceptor scaled by its molar extinction coefficient. From Figure S14a, the *J* value for the rubrene donor/**Au_42_** acceptor system was determined to be 1.784 × 10^15^ M^−1^cm^−1^nm^4^. The refractive index of toluene at room temperature is *n* = 1.496, and the Φ_D_ of concentrated rubrene (20 mM) was set to 0.76 (= Φ_F_∙Φ_out_), taking into account the effect of self-absorption under this concentration condition. Assuming isotropic dynamic averaging of the donor and acceptor in solution, the mean value of *κ*^2^ is 2/3. As a result, the *R*_0_ value for the rubrene/**Au_42_** system was calculated to be 4.7 nm. Figure S14b shows the calculated Φ_FRET_ as a function of the D–A distance *r* under this *R*_0_, using the following equation:

$$\text{Φ}_{\text{FRET}}\text{ }\text{=}\text{ }\frac{\text{1}}{\text{1}\text{ }\text{+}\text{ }\left( \text{r}/{\text{R}_{\text{0}}} \right)^{\text{6}}}\text{,}\text{ } \text{(S1}\text{5}\text{)}$$

**Supplemental data**


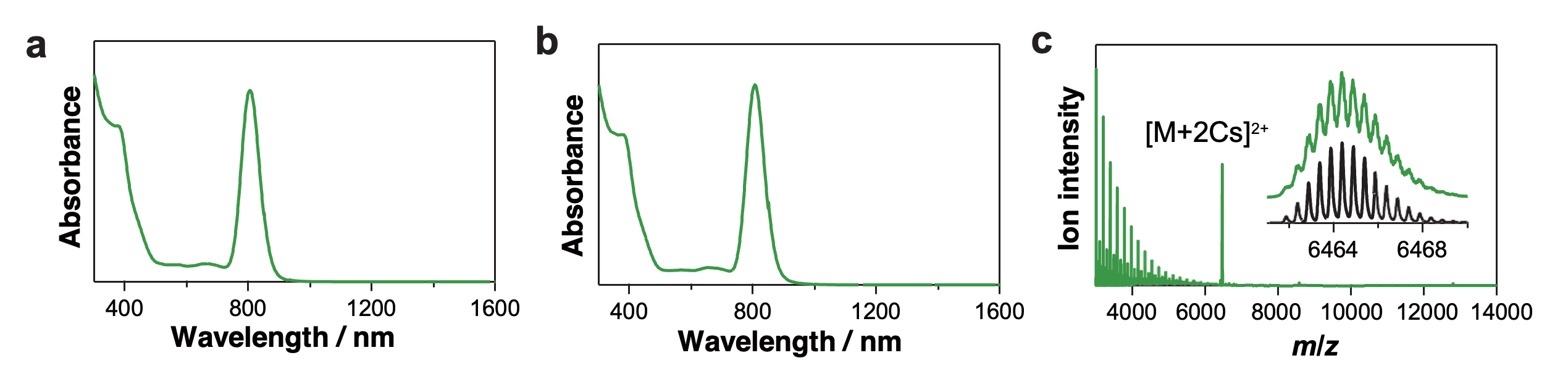


**Figure S1.** (a) Absorption spectra of **Au_42_** after PTLC separation. (b) Absorption and (c) positive mode ESI mass spectrum of **Au_42_** after GPC purification. The inset in panel (c) compares the observed (colored) and simulated (black) isotope patterns of the observed peaks.

**Figure S2.** DFT-optimized structure of Au_42_(PET)_32_ in the ground state, along with their corresponding energies and infrared spectra.

**Figure S3.** (a) Concentration-dependent absorption spectra of **Au_42_** in toluene. (b) Plot of absorbance at 807 nm as a function of **Au_42_** concentration with a corresponding linear fit.

**Figure S4.** Comparison of the transmittance curves of **Au_42_**, [Au_25_(PET)_18_]^−^, and [Au_25_(PPh_3_)_10_(PET)_5_Cl_2_]^2+^ (Au_25_-rod), normalized to the same transmittance at 808 nm (10^−^*^A^*^/2^ = 0.0363, *A* = 2.88), together with the FL spectrum of rubrene in toluene. Panel (a) shows the transmittance curves on a linear scale, while panel (b) presents the same data on a logarithmic scale.

1.0

**O1**

Current / µA

0.5

**R1**

0

Potential vs. Fc^+^/Fc / V

–0.5

**1.48 V**

–1.0

0.5

0

–0.5

–1.0

–1.5

**Figure S5.** DPV chart of **Au_42_** in 0.1 M TBAPF_6_/DCM at room temperature. Downward arrow indicates the corresponding open circuit potential. O1 (0.184 V) and R1 (–1.292 V) represent 1st oxidation and reduction peaks of **Au_42_**, respectively.

**Figure S6.** Stern–Volmer plots constructed from the photoluminescence (PH) lifetimes, with a linear fit used to determine the Stern–Volmer constant (*K*_SV_) and the triplet energy transfer rate constant (*k*_TET_).

**Figure S7.** (a) and (b) Intensity autocorrelation function, *G^(^*^2)^(*t*), of the scattered light (left), and the average particle diameter as a function of accumulation number obtained by DLS measurements for toluene solutions of **Au_42_** (16 µM) and **Au_42_** (16 µM)/rubrene (20 mM), respectively. For both solutions, no signal of the scattering autocorrelation function was observed, and the average particle diameter remained zero throughout the measurements, indicating the absence of aggregate formation.

**Figure S8.** Excitation position dependence of UC emission spectra.

**Figure S9.** Fluorescence decay profiles of a deaerated toluene solution containing **Au_42_** (6 μM) and rubrene (15 mM), recorded at 880 ± 15 nm upon 805 nm excitation, with the fitting curve (red) and instrument response function (blue).

**Figure S10.** Internal reference method for determining the upconversion (UC) quantum yield (Φ_UC_) and the internal UC quantum yield (Φ_UCg_). The area of the prompt fluorescence (FL) band of **Au_42_** was used as the internal reference. To obtain Φ_UCg_, Φ_out_ was calculated from the ratio of the measured UC spectrum area (*S*_UC_) to the fluorescence spectrum area of annihilator in a dilute solution (*S*_FL(annihilator)_). For further details, see the main text.

**Figure S11.** Example of deconvolution of the emission spectrum from **Au_42_** in a **Au_42_** (16.3 μM)/rubrene (20 mM) solution under 936 nm excitation into fluorescence (FL) and phosphorescence (PH) components using a two-Gaussian fitting approach. The area of the fluorescence component (*S*_FL_) was used as an internal reference for calculating Φ_UC_ under 936 nm excitation (see Figure S7).

**Figure S12.** Jablonski diagram depicting the generation of delayed fluorescence (FL) through triplet–triplet annihilation (TTA) between **Au_42_** NCs, initiated by the S_0_ → S_1_ hot-band transition and the S_0_ → T_1_ transition in **Au_42_**.

**Figure S13.** (a) Excitation-intensity (*I*_ex_) dependence of the emission spectra of a deaerated toluene solution containing **Au_42_** (5.2 μM) and TES-ADT (10.1 mM) under 808 nm excitation. (b) *I*_ex_ dependence of the upconversion (UC) quantum yield (Φ_UC_) for the same system. The solid line represents the fit using Equation 4. (c) Comparison of the UC spectrum of the **Au_42_**/TES-ADT solution and fluorescence (FL) spectrum of a dilute TES-ADT/toluene solution used for Φ_out_ determination. (d) *I*_ex_-dependence of the UC emission of TES-ADT and the prompt FL of **Au_42_** for the same system. The FL data points are linearly fitted, while the UC data points are fitted using Equation 5.

**Figure S14.** (a) Absorption spectrum of **Au_42_** in toluene and normalized upconverted fluorescence spectrum of rubrene in deaerated toluene containing **Au_42_** (16.3 μM) and rubrene (20 mM). The shaded region indicates the spectral overlap responsible for the overlap integral *J*. (b) Calculated FRET efficiency (Φ_FRET_) as a function of donor–acceptor distance (*r*), using a Förster radius of *R*₀ = 4.7 nm.

**Supplemental Tables**

**Table S1. Comparison of the present systems with previously reported NIR-to-visible photon upconversion performance in the liquid phase.**

| Sensitizer | | Annihilator (mM) | Solvent | *λ*_ex_ / nm | Φ_UC_ (%) (Φ_UCg_) | *I*_th_ / W cm^−2^ | *f* (%) | Ref. |
| --- | --- | --- | --- | --- | --- | --- | --- | --- |
| Metal nanoclusters | Au_42_(PET)_32_ | rubrene (20) | toluene | 808 | 16.5 (21.4) | 0.14 | 58 | This work |
|  |  | rubrene (20) | toluene | 936 | 12.3 (15.0) | 13.8 | 58 | This work |
|  |  | TES-ADT (10.1) | toluene | 808 | 4.4 (9.2) | 1.17 | 35 | This work |
|  | Au_42_(PET)_32_ | TES-ADT (10) | toluene | 808 | 3.35 | 0.09 |  | 22 |
|  | [PtAg_24_(DMBT)_18_]^2-^ | perylene (13.1) | THF | 785 | 1.1 (3.1) | >14 |  | 23 |
|  | [PtAg_24_(DMBT)_18_]^2-^ | TIPS-An (12.7) | THF | 785 | 1.2 (3.9) | 1.1 |  | 23 |
|  | [Au_25-_*_x_*Cu*_x_*(PPh₃)_10_ (PET)_5_Cl_2_]^2+^ | BPEA (20) | THF | 805 | 0.72 (2.33) | 2.9 |  | 24 |
|  | [Au_25_(PPh₃)_10_(PET)_5_Cl_2_]^2+^ | BPEA (20) | THF | 805 | 0.11 (0.49) | 2.5 |  | 24 |
| Semiconductor QDs | PbS-S (2.7 nm)/5-CT | rubrene (20) | toluene | 781 | 2.3 | – |  | 25 |
|  | PbS-T (2.7 nm)/5-CT | rubrene (20) | toluene | 781 | 5.9 | 53.4 |  | 25 |
|  | PbS/ZnS/5-CT | rubrene (20) | toluene | 785 | 0.14 | – |  | 26 |
|  | PbSe (2.1 nm) | rubrene (3.84) | toluene | 800 | 0.005 | ~60 |  | 27 |
|  | PbS (2.9 nm)/CPT | rubrene (20) | toluene | 808 | 0.85 |  |  | 28 |
|  | PbSe (2.5 nm)/CPT | rubrene (20) | toluene | 808 | 1.05 |  |  | 28 |
|  | PbS (2.7 nm)/CdS/5-CT | rubrene (20) | toluene | 808 | 4.2 | 0.0032 |  | 29 |
|  | AgBiS_2_ (3.0 nm)/5-TC | rubrene (20) | toluene | 808 | 5.25 | 93 |  | 30 |
|  | Zn-doped CuInSe_2_/5-TCA | rubrene (16) | toluene | 808 | 8.35 | 2.1 |  | 31 |
|  | InAs/ZnSe (~2.8 nm)/5-CT | rubrene (15) | toluene | 808 | 10.55 | 20.2 |  | 32 |
|  | PbS (2.73 nm)/Th-DPP | rubrene (20) | toluene | 808 | 3.25 (6.75) | 4.8 |  | 33 |
|  | PbS (3.11 nm)/Th-DPP | rubrene (20) | toluene | 980 | 0.10 (0.225) | 19.9 |  | 33 |
|  | PbS (3.11 nm)/Th-DPP | rubrene (20) | toluene | 1064 | 0.08 (0.185) | 23.5 |  | 33 |
|  | PbS | TES-ADT (50) | toluene | 1064 | 0.047 | 43 |  | 34 |
|  | PbS/TTCA | V79 (2) | toluene | 808 | 0.031 | – |  | 35 |
| Metal complexes | PdTNP | Py5 (0.1) | toluene | 720 | 7.05 | 0.046 |  | 36 |
|  | [Os(bptpy)_2_]^2+^ | TTBP (20) | DMF | 724 | 1.35 | 0.32 |  | 37 |
|  | [Os(peptpy)_2_]^2+^ | TTBP (20) | DMF | 724 | 2.95 | – |  | 38 |
|  | [Os(tpy)_2_]^2+^ | (*i*-Pr_2_SiH)_2_An (0.5) | THF | 724 | 5.5 | – |  | 39 |
|  | [Os(bptpy)_2_]^2+^ | BPEA cyclophane (2) | DMF | 730 | 0.1 | 0.24 |  | 40 |
|  | PdPc | rubrene (18) | toluene | 730 | 5.7 | 1.9 | 15.5 | 41 |
|  | PtPc | rubrene (18) | toluene | 730 | 4.9 | 11.0 |  | 41 |
|  | PdPc | 2CN-Rub (18) | toluene | 730 | 2.4 | 22 | 17 | 42 |
|  | PdPc | 4CN-Rub (18) | toluene | 730 | 2.6 | 14 | 17 | 42 |
|  | PdPc | TIPS-BTX (0.39) | toluene | 730 | 3.3 | 37 |  | 43 |
|  | Texaphyrin | rubrene (0.33) | DCM | 750 | 0.77 | ~5 |  | 44 |
|  | Pyr_1_RuPZn_2_ | PDI (0.42) | MTHF | 780 | 0.375 | 0.022 |  | 45 |
|  | PtTPBP | TIP-Ac (1) | toluene | 785 | 2.1 | ~300 |  | 46 |
|  | PtNac | TDI (0.6) | DCB | 856 | 0.0089 | >30 |  | 47 |
|  | PdNac | TDI (0.6) | DCB | 856 | 0.067 |  |  | 47 |
|  | PdTAP | rubrene (1) | toluene | 785 | 1.2 | – |  | 48 |
|  | [Os(bip)_2_]^2+^ | BPEACOO^-^ (0.05) | THF | 785 | 13 | 0.0059 |  | 49 |
| Organic radicals | D1 | rubrene (5) | DCM | 938 | 0.00235 | 198 |  | 50 |
|  | TTM-TPA | perylene (8) | toluene | 733 | 6.8 | 0.51 | 86 | 51 |
|  |  | rubrene (15) | toluene |  | 2.7 | 0.94 | 29 | 51 |

**Table S2. Fluorescence (FL) and phosphorescence (PH) lifetimes and corresponding quantum yields of Au_42_ and rubrene in deaerated toluene.**

| Sample | *τ*_FL_ / ns | Φ_FL_ (%) *^a^* | *τ*_PH_ / µs | Φ_PH_ (%) *^b^* |
| --- | --- | --- | --- | --- |
| **Au_42_** (6 µM) | 0.56 | 5.0 | 2.5 | 2.3 |
| **Au_42_** + rubrene (15 mM) | 0.56 | 5.2 | 0.42 | - |

*^a^* Determined from absolute quantum yield measurements. *^b^* Determined from the relative method.

**Table S3. Gibbs free energy change of charge transfer between Au_42_ and aromatic molecules in toluene estimated using the Rehm–Weller equation.*^a^***

| Donor  (D) | Acceptor (A) | *R*_D_ / Å *^b^* | *R*_A_ / Å *^b^* | *ε*_ref_ *^c^* | *ε*_s_ *^c^* | $\text{E}_{\text{D}}^{\text{ox}}$ / V  *vs* NHE | $\text{E}_{\text{A}}^{\text{red}}$ / V *vs* NHE | *E*_T_ / eV | *C* / eV | Δ*G*_CT_ / eV |
| --- | --- | --- | --- | --- | --- | --- | --- | --- | --- | --- |
| **Au_42_** | BPEA | 10.62 | 4.46 | 8.93 | 2.38 | 0.84 | −1.52 *^d^* | 1.18 | 0.31 | +1.49 |
| BPEA | **Au_42_** |  |  |  |  | 1.05 *^d^* | −0.64 |  |  | +0.81 |
| **Au_42_** | TIPS- tetracene |  | 5.32 |  |  | 0.84 | −1.34 *^e^* |  | 0.25 | +1.31 |
| TIPS- tetracene | **Au_42_** |  |  |  |  | 0.96 *^e^* | −0.64 |  |  | +0.72 |
| **Au_42_** | rubrene |  | 4.95 |  |  | 0.84 | −1.44 *^f^* |  | 0.27 | +1.40 |
| rubrene | **Au_42_** |  |  |  |  | 0.98 *^f^* | −0.64 |  |  | +0.75 |
| **Au_42_** | TES-ADT |  | 5.12 |  |  | 0.84 | −1.35 *^g^* |  | 0.26 | +1.32 |
| TES-ADT | **Au_42_** |  |  |  |  | 0.80 *^g^* | −0.64 |  |  | +0.56 |

*^a^* Δ*G*_CT_ and *C* were calculated using Equations S4 and S5, respectively. *^b^* The van der Waals radius was estimated from optimized ground state structures obtained by density functional theory calculations at the PBE0 functional for **Au_42_** and at the M06-2X/6-31G(d,p) level for the aromatic acceptors. *^c^* *ε*_ref_ and *ε*_s_ are the refractive indices of dichloromethane and toluene, respectively. *^d^* Reference [52]. *^e^* Reference [53]. *^f^* Reference [54]. *^g^* Reference [55].

| **Table S4**. **Phosphorescence quenching parameters of Au_42_ obtained with four aromatic acceptors in deaerated toluene (*λ*_ex_ = 634 nm).** | | | | |
| --- | --- | --- | --- | --- |
| Donor | Acceptor | *E*_T_ / eV | *K*_SV_ / M^–1^ | *k*_TET_ / M^–1^ s^–1^ |
| **Au_42_** | BPEA | 1.30 | 4.5 | 1.09 × 10^6^ |
|  | TIPS-Tc | 1.21 | 154 | 6.16 × 10^7^ |
|  | rubrene | 1.14 | 313 | 1.25 × 10^8^ |
|  | TES-ADT | 1.07 | 428 | 1.71 × 10^8^ |

**References**

1. M. Strohalm, D. Kavan, P. Novák, M. Volný, V. Havlíček, *Anal. Chem.* **2010**, *82*, 4648–4651.
2. L. Luo, Z. Liu, J. Kong, C. G. Gianopoulos, I. Coburn, K. Kirschbaum, M. Zhou, R. Jin, *Proc. Natl. Acad. Sci. U.S.A.* **2024**, *121*, e2318537121.
3. M. J. Frisch, G. W. Trucks, H. B. Schlegel, G. E. Scuseria, M. A. Robb, J. R. Cheeseman, G. Scalmani, V. Barone, G. A. Petersson, H. Nakatsuji, *et al.*, *Gaussian 16, Revision B.01*, Gaussian, Inc., Wallingford CT, **2016**.
4. J. Perdew, M. Ernzerhof, K. Burke, *J. Chem. Phys.* **1996**, *105*, 9982–9985.
5. F. Weigend, R. B. Ahlrichs, *Phys. Chem. Chem. Phys*. **2005**, *7*, 3297–3305.
6. M. M. Francl, W. J. Pietro, W. J. Hehre, J. S. Binkley, M. S. Gordon, D. J. DeFrees, J. A. People, *J. Chem. Phys.* **1982**, *77*, 3654–3665.
7. D. Andrae, U. Häußermann, M. Dolg, H. Stoll, H. Preuß, *Theor. Chim. Acta* **1990**, *77*, 123.
8. T. Yanai, D. P. Tew, N. C. Handy, *Chem. Phys. Lett.* **2004**, *393*, 51–56.
9. Y. Zhao, D. G. Truhlar, Theor. Chem. Acc. 2008, *120*, 215−241.
10. Y. Niihori, Y. Wada, M. Mitsui, *Angew. Chem., Int. Ed*. **2021**, *60*, 2822–2827.
11. D. Arima, Y. Niihori, M. Mitsui, *J. Mater. Chem. C*, **2022**, *10*, 4597−4606.
12. M. Mitsui, A. Uchida, *Nanoscale*, **2024**, *16*, 3053−3060.
13. M. Mitsui, Y. Miyoshi, D. Arima, *Nanoscale* **2024**, *16*, 14757–14765.
14. T. Nakagawa, K. Okamoto, H. Hanada, R. Katoh, *Opt. Lett*. **2016**, *41*, 1498–1501.
15. G. Orellana, A. Braun, *J. Photochem. Photobiol. A : Chem.* **1989**, *48*, 277−289.
16. J. J. Snellenburg, S. P. Laptenok, R. Seger, K. M. Mullen, I. H. M. van Stokkum, *Journal of Statistical Software*, **2012**, *49*(3), 1–22.
17. M. Mitsui, D. Arima, A. Uchida, K. Yoshida, Y. Arai, K. Kawasaki, Y. Niihori, *J. Phys. Chem. Lett.* **2022**, *13*, 9272−9278.
18. S. M. Bachilo, R. B. Weisman, *J. Phys. Chem. A* **2000**, *104*, 7711–7714.
19. S. Prathapan, S. L. Yang, J. Seth, M. A. Miller, D. F. Bocian, D. Holten, J. S. Lindsey, *J. Phys. Chem. B* **2001**, *105*, 8237–8248.
20. B. Valeur, Molecular Fluorescence: Principles and Applications; Wiley-VCH: Weinheim, Germany, 2012.
21. Y. Zhou, F. N. Castellano, T. W. Schmidt, K. Hanson, *ACS Energy Lett.* **2020**, *5*, 2322–2326.
22. Z. Liu, X. Hu, L. Luo, G. He, A. Mazumder, E. Gunay, Y. Wang, E. C. Dickey, L. A. Peteanu, K. Matyjaszewski, R. Jin, *J. Am. Chem. Soc.* **2025**, *147*, 28241−28250.
23. Y. Niihori, Y. Wada, M. Mitsui, *Angew. Chem., Int. Ed.* **2021**, *60*, 2822−2827.
24. M. Mitsui, Y. Miyoshi, D. Arima, *Nanoscale* **2024**, *16*, 14757−14765.
25. Z. Huang, Z. Xu, M. Mahboub, Z. Liang, P. Jaimes, P. Xia, K. R. Graham, M. L. Tang, T. Lian, *J. Am. Chem. Soc*. **2019**, *141*, 9769−9772.
26. M. Mahboub, P. Xia, J. V. Baren, X. Li, C. H. Lui, M. L. Tang, *ACS Energy Lett*. **2018**, *3*, 767−772.
27. Z. Huang, X. Li, M. Mahboub, K. M. Hanson, V. M. Nichols, H. Le, M. L. Tang, C. J. Bardeen, *Nano Lett*. **2015**, *15*, 5552.
28. Z. Huang, D. E. Simpson, M. Mahboub, X. Li, M. L. Tang, *Z. Chem. Sci.* **2016**, *7*, 4101−4104.
29. M. Mahboub, Z. Huang, M. L. Tang, *Nano Lett*. **2016**, *16*, 7169−7175.
30. K. T. Chang, W. Liang, S. Gong, P. H. Yeung, J. Feng, X. Chen, H. Lu, *J. Am. Chem. Soc*. **2025**, *147*, 14015–14023.
31. W. Liang, C. Nie, J. Du, Y. Han, G. Zhao, F. Yang, G. Liang, K. Wu, *Nature* *Photonics* **2023**, *17*, 346–353.
32. R. Sun, J. Zang, R. Lai, W. Yang, B. Ji, *J. Am. Chem. Soc*. **2024**, *146*, 17618−17623.
33. L. H. Jiang, X. Miao, M. Y. Zhang, J. Y. Li, L. Zeng, W. Hu, L. Huang, D. W. Pang, *J. Am. Chem. Soc.* **2024**, *146*, 10785–10797.
34. N. Nishimura, J. R. Allardice, J. Xiao, Q. Gu, V. Gray, A. Rao, *Chem. Sci*. **2019**, *10*, 4750−4760.
35. E. M. Gholizadeh, S. K. K. Prasad, Z. L. Teh, T. Ishwara, S. Norman, A. J. Petty, J. H. Cole, S. Cheong, R. D. Tilley, J. E. Anthony, S. Huang, T. W. Schmidt, *Nat. Photonics* **2020**, *14*, 585–590.
36. L. Huang, W. Wu, Y. Li, K. Huang, L. Zeng, W. Lin, G. Han, *J. Am. Chem. Soc.* **2020**, *143*,18460−18470.
37. Y. Sasaki, S. Amemori, H. Kouno, N. Yanai, N. Kimizuka, *J. Mater. Chem. C* **2017**, *5*, 5063−5067.
38. Y. Sasaki, M. Oshikawa, P. Bharmoria, H. Kouno, A. Hayashi-Takagi, M. Sato, I. Ajioka, N. Yanai, N. Kimizuka, *Angew. Chem., Int. Ed.* **2019**, *58*, 17827−17833.
39. R. Haruki, Y. Sasaki, K. Masutani, N. Yanai, N. Kimizuka, *Chem. Commun.* **2020**, *56*, 7017−7020.
40. K. Mase, Y. Sasaki, Y. Sagara, N. Tamaoki, C. Weder, N. Yanai, N. Kimizuka, *Angew. Chem., Int. Ed.* **2018**, *57*, 2806−2810.
41. E. Radiunas, S. Raišys, S. Juršėnas, A. Jozeliūnaitė, T. Javorskis, U. Šinkevičiūtė, E. Orentas, K. Kazlauskas, *J. Mater. Chem. C* **2020**, *8*, 5525−5534.
42. E. Radiunas, L. Naimovičius P. Baronas, A. Jozeliūnaitė, E. Orentas, K. Kazlauskas, *Adv. Opt. Mater.* **2025**, 2403032.
43. A. T. Gilligan, R. Owens, E. G. Miller, N. F. Pompetti, N. H. Damrauer, *Chem. Sci*. **2024**, *15*, 1283−1296.
44. F. Deng, W. Sun, F. N. Castellano, *Photochem. Photobiol. Sci.* **2014**, *13*, 813−819.
45. T. N. Singh-Rachford, A. Nayak, M. L. Muro-Small, S. Goeb, M. J. Therien, F. N. Castellano, *J. Am. Chem. Soc*. **2010**, *132*, 14203–14211.
46. N. Nishimura, V. Gray, J. R. Allardice, Z. Zhang, A. Pershin, D. Beljonne, A. Rao, *ACS. Materials. Lett.* **2019**, *1*, 660−664.
47. S. Amemori, N. Yanai, N. Kimizuka, *Phys. Chem. Chem. Phys.* **2015**, *17*, 22557–22560.
48. V. Yakutkin, S. Aleshchenkov, S. Chernov, T. Miteva, G. Nelles, A. Cheprakov S. Baluschev, *Chem. – Eur. J*. **2008**, *14*, 9846–9850.
49. J. H. Baek, D. Song, G. Park, S. Avula, S. Park, K. Ohkubo, Y. You, *Adv. Opt. Mater.* **2025**, *13*, 2500876.
50. S. Amemori, Y. Sasaki, N. Yanai, N. Kimizuka, *J. Am. Chem. Soc.* **2016**, *138*, 8702–8705.
51. Y. Wei, K. An, X. Xu, Z. Ye, X. Yin, X. Cao, C.Yang, *Adv. Opt. Mater.* **2024**, *12*, 2301134.
52. V. Gray, A. Dreos, P. Erhart, B. Albinsson, K. Moth-Poulsen, M. Abrahamsson, *Phys. Chem. Chem. Phys.* **2017**, *19*, 10931–10939.
53. O. L. Griffith, A. G. Jones, J. E. Anthony, D. L. Lichtenberger, *J. Phys. Chem. C* **2010**, *114*, 32, 13838–13845.
54. F. Anger, T. Breuer, A. Ruff, M. Klues, A. Gerlach, R. Scholz, S. Ludwigs, G. Witte, F. Schreiber, *J. Phys. Chem. C* **2016**, *120*, 10, 5515–5522.
55. K. J. Thorley, H. Le, Y. Song, J. E. Anthony, *J. Mater. Chem. C*, **2022**, *10*, 15861-15871.
